# Identification and analysis of *Eimeria nieschulzi* gametocyte genes reveal splicing events of *gam* genes and conserved motifs in the wall-forming proteins within the genus *Eimeria* (Coccidia, Apicomplexa)

**DOI:** 10.1051/parasite/2017049

**Published:** 2017-12-06

**Authors:** Stefanie Wiedmer, Alexander Erdbeer, Beate Volke, Stephanie Randel, Franz Kapplusch, Sacha Hanig, Michael Kurth

**Affiliations:** Institute for Zoology, Technische Universität Dresden, Helmholtzstraße 10, 01062 Dresden Germany

**Keywords:** *Eimeria nieschulzi*, gametocyte, GAM, alternative splicing, polymorphism

## Abstract

The genus *Eimeria* (Apicomplexa, Coccidia) provides a wide range of different species with different hosts to study common and variable features within the genus and its species. A common characteristic of all known *Eimeria* species is the oocyst, the infectious stage where its life cycle starts and ends. In our study, we utilized *Eimeria nieschulzi* as a model organism. This rat-specific parasite has complex oocyst morphology and can be transfected and even cultivated *in vitro* up to the oocyst stage. We wanted to elucidate how the known oocyst wall-forming proteins are preserved in this rodent *Eimeria* species compared to other *Eimeria*. In newly obtained genomics data, we were able to identify different gametocyte genes that are orthologous to already known *gam* genes involved in the oocyst wall formation of avian *Eimeria* species. These genes appeared putatively as single exon genes, but cDNA analysis showed alternative splicing events in the transcripts. The analysis of the translated sequence revealed different conserved motifs but also dissimilar regions in GAM proteins, as well as polymorphic regions. The occurrence of an underrepresented *gam56* gene version suggests the existence of a second distinct *E. nieschulzi* genotype within the *E. nieschulzi* Landers isolate that we maintain.

## Abstract (fr)

Le genre *Eimeria* (Apicomplexa, Coccidia) fournit une large variété de différentes espèces avec différents hôtes pour étudier les caractéristiques communes et variables dans le genre et ses espèces. Une caractéristique commune de toutes les espèces connues d'*Eimeria* est l'oocyste, l'étape infectieuse par laquelle son cycle de vie commence et se termine. Dans notre étude, nous avons utilisé *Eimeria nieschulzi* comme organisme modèle. Ce parasite spécifique au rat présente une morphologie complexe de ses oocystes et peut être transfecté et même cultivé *in vitro* jusqu'au stade oocyste. Nous voulions élucider comment, dans cette espèce d'*Eimeria* de rongeurs, les protéines de formation de paroi d'oocystes connues sont préservées, en comparaison aux autres *Eimeria*. Dans les nouvelles données génomiques obtenues, nous avons pu identifier différents gènes de gamétocytes qui sont orthologues aux gènes *gam* déjà connus et impliqués dans la formation de la paroi d'oocystes d'espèces d'*Eimeria* aviaires. Ces gènes apparaissent possiblement comme des exons isolés, mais l'analyse par ADNc a montré des événements d'épissage alternatifs dans les transcripts. L'analyse de la séquence traduite a révélé différents motifs conservés et aussi des régions dissemblables dans les protéines GAM, ainsi que des régions polymorphes. L'existence d'une version sous-représentée du gène *gam56* suggère l'existence d'un deuxième génotype distinct d'*E. nieschulzi* dans l'isolat *E. nieschulzi* Landers que nous entretenons.

## Introduction

Coccidian parasites share the common persistent stage, the oocyst. The uptake of an oocyst and hatching of the infectious sporozoite stage in the host marks the beginning of the parasite's development within the host. After asexual and sexual reproduction, the formation of the oocyst initiates the end of the development in the host. Coccidian parasites of the genus *Eimeria* have monoxenous life cycles, whereas *Toxoplasma gondii* and *Sarcocystis* spp. have intermediate hosts and show some variations in their development. Oocyst composition and formation have been examined for representative species and in multiple studies [3–8,10,14–16,19,21,28,35,38–40,47–48].

The oocyst wall is formed by the so-called wall-forming bodies. The electron-dense wall-forming bodies (WFBI), present in the macrogamont, form the outer wall, and the less electron-dense, textured wall-forming bodies (WFBII) form the inner wall [37]. The dominant proteins in *Eimeria maxima* macrogamonts were described with a molecular weight of 56 kDa and 82 kDa [46] and were found in the WFBII [3–4,16].

In *E. maxima* and *E. tenella,* two variants of *gam56* have been described [4,22], while *gam82* is unique [6].

These GAM proteins, encoded by single exon *gam* genes, harbour tyrosine-rich amino acid motifs that are involved in crosslinking of proteins via dityrosine bonds and a hardening process, indicated by a blue autofluorescence of the oocyst wall [5–6]. During wall formation, the GAM proteins are processed into smaller fragments [5,32]. The exact mechanism of crosslinking remains unknown, but it is assumed that peroxidases play a role in this process [29]. Recent publications also show upregulation of oxidoreductase, as well as oxidases during wall formation, implying that these enzymes might play a role in the crosslinking process [44]. The blue autofluorescence can also be observed in other *Eimeria* species [7,13,19] and other coccidian parasites like *Toxoplasma gondii* and *Isospora* [13,26]. Avian *Eimeria* species are one of the groups within the genus that have been investigated in the greatest detail concerning oocyst wall formation because of their economic importance. In contrast, rodent *Eimeria* species have lower economic importance but serve as interesting model organisms in immunology and cell biology [21,43]. However, they differ in some aspects to avian *Eimeria* species concerning oocyst morphology and excystation [48].

Within avian *Eimeria*, GAM proteins are highly conserved [8]. We wanted to scrutinize the extent of conservation of these proteins, and determine which features are shared by both groups and which are unique. To examine these questions we analysed genome and cDNA data for the rat-specific parasite *Eimeria nieschulzi.*

## Material and methods

### Ethics

The authors declare that the experiments comply with the current laws of Germany, where they were performed. Experiments in animals were registered at Regierungspräsidium Dresden (Reference Numbers 24–9168.25–8–2004–1).

### Parasites

*Eimeria nieschulzi* was propagated in rats (*Rattus norvegicus*, Sprague Dawley^®^ Rat, Crl:SD) as previously described [21]. For gametocyte production, infected rats were euthanised at 149-153 hours post-infection (h p.i.).

### Purification of macrogametocytes of *E. nieschulzi* (modified after [32,34])

**Figure 1:**
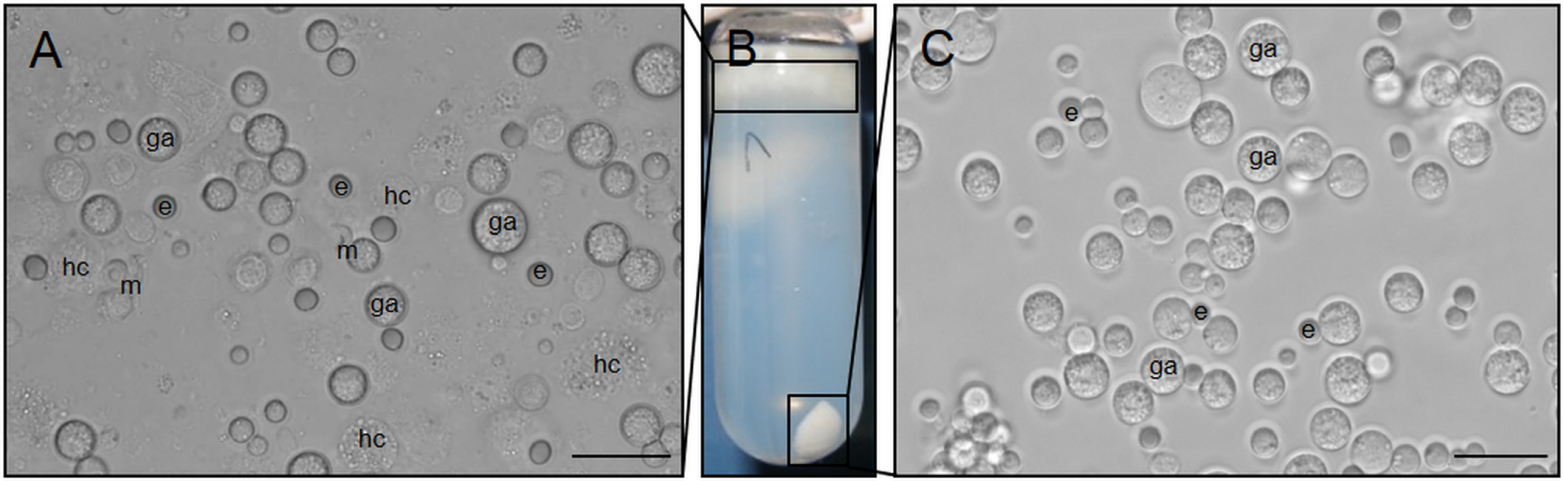
Light micrographs of macrogametocytes of *E. nieschulzi* (149 h p.i.) after Percoll density-gradient centrifugation. **A** Upper gradient fraction contained gametocytes (ga), rat small intestine cells (hc) and erythrocytes (e). **B** Discontinuous Percoll gradient. **C** Lower gradient fraction contained purified macrogametocytes (ga) and erythrocytes (e). e erythrocyte, ga gametocyte, hc host cell, m merozoite of fourth generation. Bar: 20 μm.

Rats (*Rattus norvegicus*, Sprague Dawley^®^ Rat, Crl:SD) were orally infected by gavage with 500,000 sporulated oocysts of *E. nieschulzi* and euthanised at 149-153 h p.i. The small intestine was removed, dissected, cut open lengthwise and washed with ice-cold Dulbecco's Medium (DM). The gametocytes containing mucosa were scraped with a glass slide. The scrapings were resuspended in DM, centrifuged (2x, 10 min, 800 xg) and filtered using three filters of different sizes, starting with the largest ones (metallic filter, mesh size: 500 µm and 400 µm) and ending with the smallest one (mesh size: 70 µm (Fisherbrand^®^; Thermo Fisher Scientific Inc.)). To prepare an isotonic stock solution of Percoll (GE Healthcare Life Sciences), Percoll was diluted 10:1 with 1.5 M NaCl, according to the manufacturer's instructions. The isotonic stock solution was diluted to lower densities (30, 50, 60% Percoll) by adding 0.15 M NaCl. The gametocytes were concentrated and purified by centrifugation on a discontinuous Percoll gradient (Figure 1B) at 400 xg for 15 min at 4 °C (Avanti^®^ J-26 XP, Rotor Beckman JA25.50, Beckman Coulter, Inc.), according to Mouafo et al. (2002) [32]. For further experiments, Percoll was removed by washing with PBS and centrifugation (3x).

### Genomic DNA extraction

Sporozoites of sporulated, sodium hypochlorite-treated oocysts were excysted as published previously [23] and purified by nylon wool/DEAE cellulose [36,41]. Lysis of sporozoites (10^9^) was performed with a NucleoSpin^®^ Tissue Kit (Macherey-Nagel GmbH & Co. KG), according to the manufacturer's recommendations. Purification of genomic DNA was carried out with phenol-chloroform extraction [33]. DNA was quantified using a NanoDrop 1000 Spectrophotometer.

### Sequencing and assembly of genomic DNA

Approx. 10 μg of the genomic DNA was sent to GATC Biotech AG (Konstanz), which performed sample preparation and Illumina Hiseq2000 sequencing (Paired-end) with a read length of 100 bp. The genomic assembly was performed by GATC Biotech AG with CLC Workbench.

### BLAST analysis

Based on the already published and characterised gametocyte antigens (GAM56/82) from *E. maxima* (GenBank Accession No. CDJ56915, CDJ56914, XP_013333566), BLAST search on the *E. nieschulzi* genomic assembly (Accession No. JRZD00000000) was performed via a TBLASTN search. Contigs potentially encoding GAM protein sequences were analysed for open reading frames with pDRAW32 software (acaclone.com). Amino acid sequences of ORFs were aligned with *E. maxima* gametocyte protein sequences (GAM56/82).

### RNA extraction, cDNA synthesis and library construction

RNA from purified gametocytes was isolated using the High Pure RNA Isolation Kit (Roche Molecular Systems Inc.). The cDNA synthesis was carried out using the Maxima H Minus First Strand cDNA Synthesis Kit (Thermo Fisher Scientific Inc.) with Oligo dT_15_ Primer (#1) or the GeneRacer OligodT Cloned AMV RT Module (Invitrogen, #2). The cDNA was purified from the synthesis assay using the GeneJET Gel Extraction Kit (Thermo Fisher Scientific Inc.) and eluted in distilled H_2_O.

For cDNA library construction, the mRNA was enriched from total RNA with the GenElute™ mRNA Miniprep Kit (Sigma-Aldrich^®^). First and second strand synthesis was performed with the cDNA synthesis Kit (Roche Molecular Systems Inc.), according to the manufacturer's recommendations. The cDNA was purified with the GeneJET Gel Extraction Kit (Thermo Fisher Scientific Inc.) and eluted in 25 μl distilled H_2_O. The purified cDNA was directly cloned into the pJET1.2 plasmid via the CloneJET PCR Cloning Kit (Thermo Fisher Scientific Inc.) and introduced by heat shock in NEB 10 beta competent *E. coli* (high efficiency), according to the manufacturer's description (New England Biolabs). Transformed *E. coli* were grown in selective LB_amp_ media overnight. Aliquots of 10 ml (with 10% glycerine) were stored at −80 °C.

### Library analysis

Dilutions (1:10) of the library were plated on LB_amp_ agar to obtain distinct colonies. Minipreps of single colonies were prepared with the GeneJET Plasmid Miniprep Kit (Thermo Fisher Scientific Inc.), according to the manufacturer's instruction. Plasmid DNA was digested with *Bgl*II and analysed by agarose gel separation. Plasmids with insert sizes of 500 bp and above were sequenced (GATC Biotech AG). The sequences were analysed by BLAST on NCBI and Genedb.org.

### Polymerase chain reaction (PCR) and rapid amplification of cDNA ends (RACE) PCR

**Figure 2:**
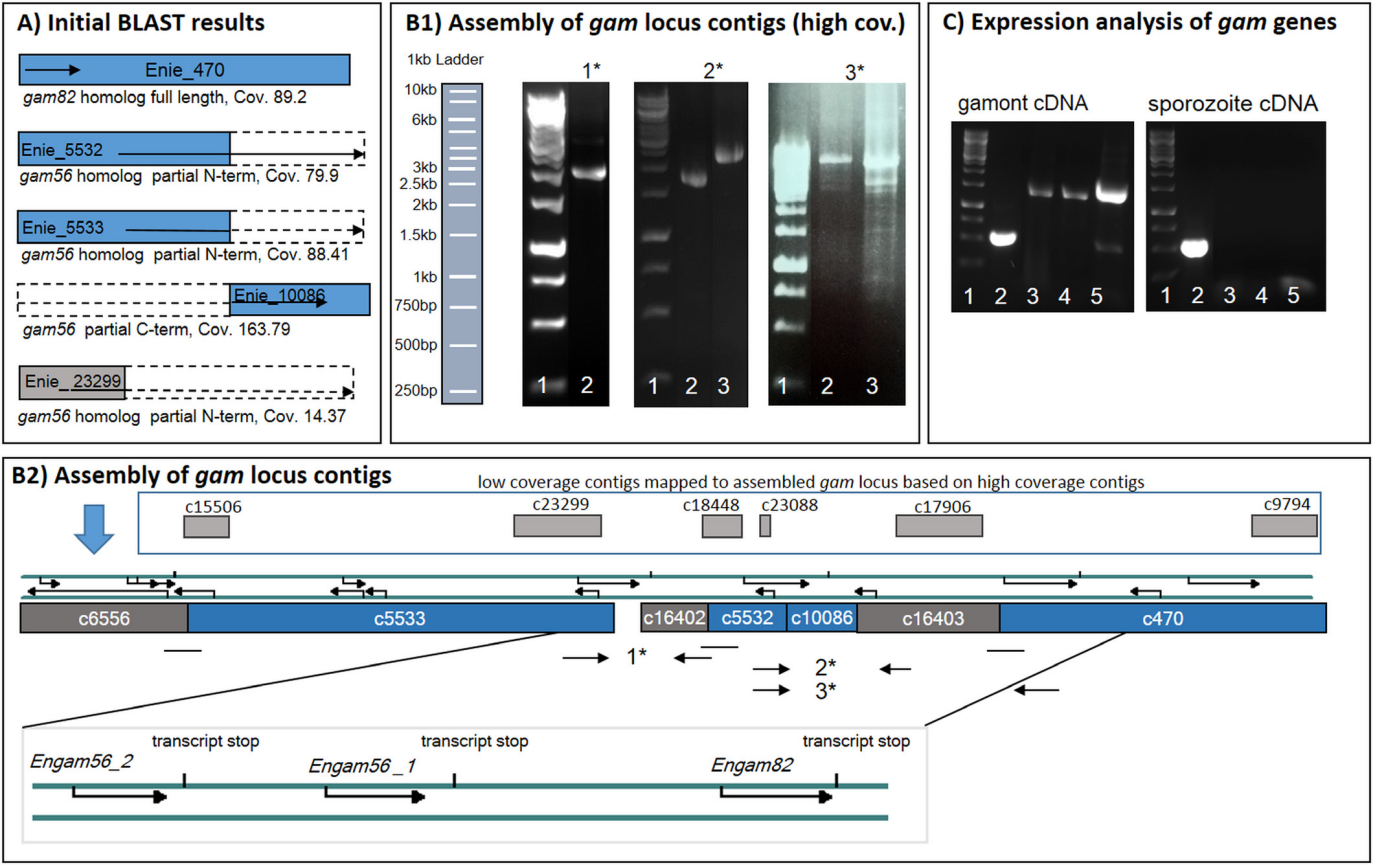
Assembly of *gam* contigs and expression analysis. **A**) Graphical Illustration of BLAST results in the *Eimeria nieschulzi* genome data. Contig Enie_470 encodes the complete *gam82* ortholog, whereas contigs Enie_5532, Enie_5533, and Enie_23299 encode only 5' parts and contig Enie_10086 encodes a 3' area of *gam56*. Dashed areas of the boxes indicate the missed sequences of the corresponding genes. Sequences were completed, ordered and manually assembled, resulting in the map shown in **B2**). **B1-2**) Mapping of contigs for final assembly. Based on the initial BLAST analysis (**A**) PCR experiments (**B1**) with potential primer pairs were performed on genomic DNA (1*-3*). 1*: PCR product (lane 2) at ca. 2,100 bp (primer #3/#7) show that contig Enie_5533 is adjacent to contig Enie­_16402. 2*: PCR products at 1,800 bp (primer #4/#5) and 2,500 bp (primer #4/#6) show vicinity of contig Enie_5532 and contig Enie_16403. 3*: PCR products at ∼8 kb (primer #8/#10 (lane 2), primer #8/#9 (lane 3) respectively, show interconnection between contig Enie_5532 and contig Enie_470 (**B2**). PCR products were verified by sequencing. Several other low-coverage contigs (Enie_23088 cov. 10.19; Enie_18448 cov. 13.62; Enie_17906 cov. 15.53; Enie_9794 cov. 15.48) map to different areas of the *gam* locus, predominantly outside of open reading frame-containing regions (**B2**). DNA size standard: 1 kb ladder. **C**) The expression of *gam* genes in gametocytes was confirmed by amplification from gametocyte cDNA with gene-specific primers (lane 2: reaction control (ribosomal subunit); lane 3: *gam56_1*; lane 4: *gam56_2*; lane 5: *gam82*; size standard lane 1: 1 kb ladder). *Gam* transcripts were absent in sporozoite cDNA (control).

PCR was performed with Biometra Personal Cycler, or Biometra TGradient and Kapa HiFi DNA polymerase (Kapa Biosystems) respectively, according to the manufacturer's recommendations. Gene-specific or contig-specific primers (for further details see Table 1) were used together with genomic DNA or cDNA, respectively. RACE-PCR was performed with gene-specific forward primers (#3; #4) and RACE reverse primers on RACE-cDNA (Table 1). For sequence analysis, PCR products were cloned into pJET1.2 plasmid (CloneJET PCR Cloning Kit, Thermo Fisher Scientific Inc.) and were analysed for expected insert size by restriction with the *Bgl*II enzyme. Inserts were sequenced afterwards by GATC Biotech AG using pJet1.2 forward or reverse primers, respectively (see Table 1). The product of the long-range PCR (Figure 2, B1, 3*) was sequenced directly with the forward primer used in the reaction.

**Table 1:** Primer sequences.

No	Name	Sequence	Annealing Temp. °C
#1	OligodT_15_	(T)_15_	50
#2	Cloned AMV RT Module	GCTGTCAACGATACGCTACGTAACGGCATGACAGTG(T)-18	42
#3	gam56_2fwd	ATGACTCGCCTCAGCCTGTG	61
#4	gam56_1fwd	ATGGTTCGTCTTATCCTTTCC	61
#5	16403intrv	AGAATCTGAT AAACAAGC	55
#6	16403exrv	AACCTTTCTA GCCGTCTTTAG	61
#7	16402exrv	TCGTGCTTGG ACATAATCGG	61
#8	EnGam56_1_fwd2	GTAAAATTCGTACCCAAGCC	55
#9	nestintEngam82reverse	GTAGCTGGAG TAACCATAAA ACGGG	55
#10	intEngam82reverse	CTGCGTTGTC CATGCCTAAG GG	55
#11	EnGam56 rv	TTATTTAGGACCCCAGGTGTATACACC	61
#12	EnGam82 fwd	CTGCCCACTCTGGAAAATGC	61
#13	EnGam82 rv	GTTGTAGGTCGTTTCCCAGG-	61
#14	RibSUfwd	AACCTGGTTGATCCTGCCAG	55
#15	RibSUrv	GGTTTTACATTCCCATCATTCC	55
#16	pJetfwd	CGACTCACTATAGGGAGAGCGGC	55
#17	pJetrv	AAGAACATCGATTTTCCATGGCAG	55

The *gam82* 5' sequence was obtained by 2-step PCR with int*Engam82*reverse (#10) and pJet primers (#16, #17) on collective library plasmid DNA as template. Products of this first PCR were used in a nested PCR with nestint*Engam82*reverse (#9) and the particular pJet primer from the previous PCR reaction. The longest obtained specific product was subcloned into pJET1.2 plasmid and subsequently sequenced (see also Supplementary File (SF)2).

Expression analysis of *gam* genes in gametocytes was performed on gametocyte-derived cDNA with gene- specific forward and reverse primers (*gam56_1*: #4/#11; *gam56_2*: #3/#11; *gam82*: #12/#13; control, ribosomal subunit: #14/#15; Table 1). Sporozoite cDNA was used as a negative control template.

### Assembly of *gam* contigs

Neighbouring contigs (high coverage) were identified by PCR with contig-specific primers (see Table 1). Sequence gaps were closed additionally by analysis of the 3' cDNA end (RACE-PCR). After preliminary arrangement of the contigs, the 5' and 3' ends of potential matching contigs were analysed for overlapping sequences, which allowed manual assembly of these contigs.

## Results

### Gametocyte purification

Macrogametocytes of *E.* *nieschulzi* were isolated and concentrated by a combination of mechanical separation, filtration and discontinuous density-gradient centrifugation, resulting in a highly pure and intact macrogametocyte fraction. After centrifugation, the fractions (Figure 1B) were collected and examined microscopically. Intracellular gametocytes, erythrocytes, rat intestinal epithelial cells and cell debris accumulated at the top of the gradient (upper band; 30% Percoll: 1.04 g/ml; Figure 1A). The lower band contained enriched isolated macrogametocytes and erythrocytes (60% Percoll: 1.075 g/ml; Figure 1C). At this juncture (149 h p.i.), the majority of parasites were macrogametocytes; only a few asexual stages (merozoites of fourth generation) could be observed (Figure 1A).

### Analysis of cDNA library and identification of GAM82 ortholog

The plasmid DNA of 120 colonies was analysed for insert size, which was at least 500 bp in 21 clones. Number and insert length of other clones: 62 clones less than 100 bp or empty vector, 37 clones between 200 and 500 bp. The sequence of only one analysed clone had similarity to mouse sequences, 20 clones had hits within Apicomplexa sequence data. The sequence of clone71 had similarity with the *E. maxima* *gam82* gametocyte antigen. Clone71 contained the 3' region of *gam82* homolog in *E. nieschulzi* (624 encoding amino acids). By PCR with pJet primer (#16/#17) and internal *gam82* reverse primer (#9/#10), the 5' region of the gene was amplified from the collective plasmid DNA of cDNA library (see SF2). Information on the cDNA 5' and 3' UTR was considered and indicated in the GenBank entry KM980455.1.

### Genome

Sequencing of the *E. nieschulzi* genomic DNA resulted in 6 × 10^7^ reads with average read length of 101 bp. Read assembly resulted in 33,467 contigs including in total 62,947,908 bp. The average contig length amounted to 1,880 bp (N75 1,683 bp, N50 5,596 bp, N25 13,548 bp). All contigs with a minimum size of 200 bp (33,146) were deposited at GenBank under the Accession No. JRZD00000000.1 (direct submission). Further analysis (performed by Emanuel Heitlinger, personal communication) showed that 21,100 contigs have coverage < 40 and 10,046 contigs have coverage > 40. BLAST analysis of contig coverage < 40 against contig coverage > 40 showed that 19,410 out of 21,100 from the Cov < 40 contigs have a hit in Cov> 40 contigs. The GenBank format does not allow coverage information; these data are provided in the supplementary material to allow discrimination of high-coverage from low-coverage sequence data (see SF3 and 4).

### Identification of *gam* genes and contig assembly

By TBLAST analysis, three contigs (Enie_5533, Enie_5532, Enie_23299) were identified encoding N-terminal sequences of the GAM56 protein, but only contig Enie_10086 encodes the C-terminal part of GAM56 (Figure 2A). Contig Enie_470 encodes the full length GAM82 (Figure 2A), which was already known from the cDNA library analysis.

The cDNA analysis of *gam56* genes showed the 3' end of the two *gam56* gene versions as nearly identical, but sequence in contig Enie_10086 is genomically associated with contig Enie_5532. A long-range PCR provided information about the association of contig Enie_5532 and contig Enie_470 (Figure 2, B1, 3*, primer #8/#10; #8/#9) and the gene flanking contig Enie_16403 (Figure 2, B1, 2*, primer #4/#5). We assumed that the similar contig Enie_16402 should be associated with the *gam56* gene version in Enie_5533, which was confirmed by PCR (Figure 2, B1, 1*, primer #3/#7). The sequential arrangement of the contigs and proximity of the sequence gaps allowed assembling the contigs encoding and flanking the *gam* genes shown in Figure 2A. The low-coverage contig Enie_23299 could not be implemented into the *gam* locus assembly, but was very similar to contig Enie_5533. Based on the cDNA analysis and other low-coverage contigs which map to the *gam* locus, predominantly outside of exon areas (Figure 2, B2), we concluded that 10% of the reads belong to a second genotype. The low-coverage contig thus represents the most contrasting sequences between both genotypes.

The *gam* genes had been designated as *gam56_1* (contig Enie_5532 and Enie_10086), *gam56_2* (contig Enie_5533) and *gam82* (contig Enie_470).

The expression of *Engam56* and *Engam82* genes in gametocytes was confirmed by analysis of gametocyte cDNA in comparison to sporozoite cDNA. All investigated *gam* genes were amplified from gametocyte cDNA, but were absent in sporozoite cDNA. A ribosomal subunit gene was amplified from both template cDNAs, indicating the existence of template DNA and a successful PCR reaction (Figure 2C).

### Analysis of *gam* genes and encoded amino acid sequences

**Figure 3:**
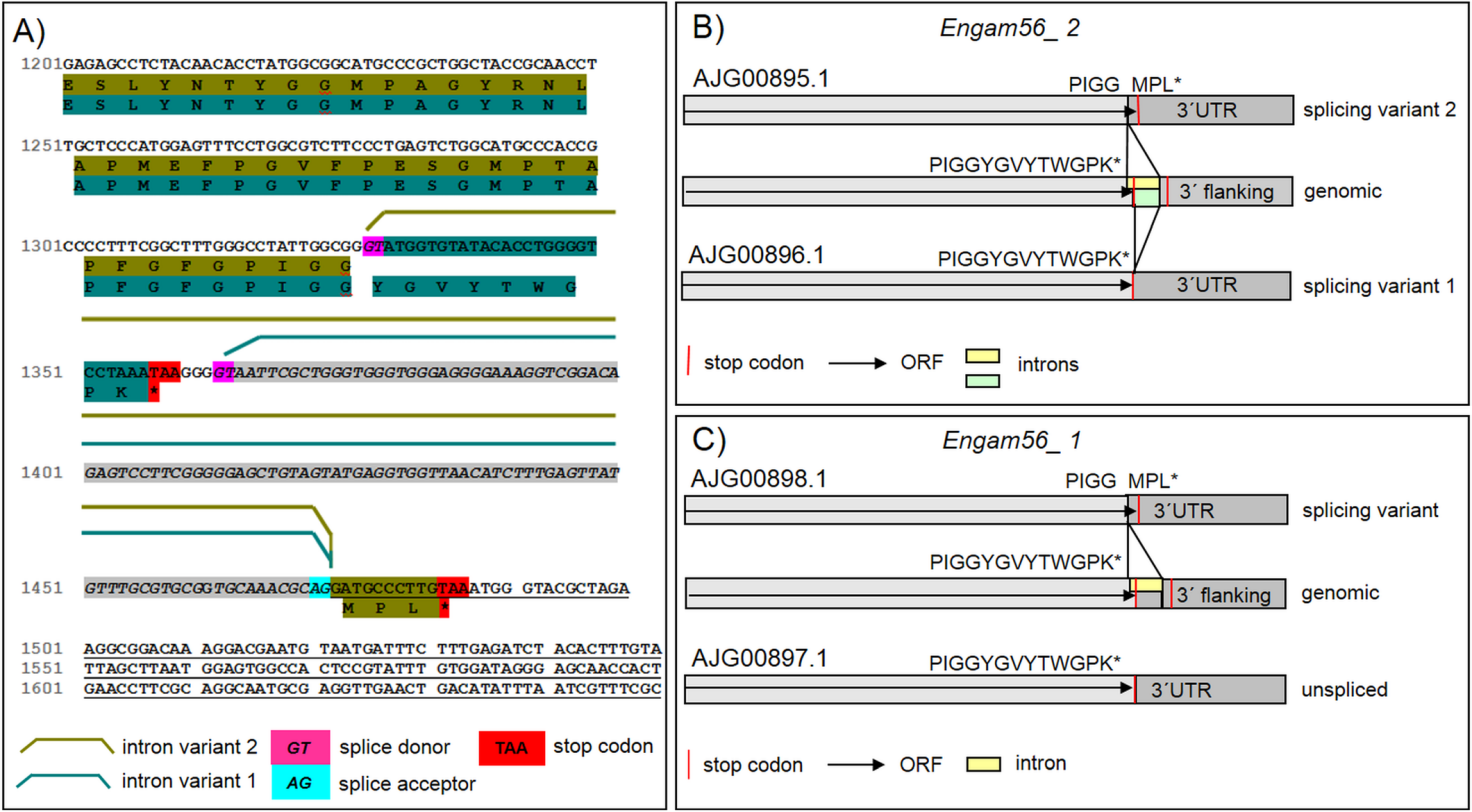
Splicing of *E. nieschulzi* *gam56* genes. **A**) Detailed view on the splicing of *Engam56_2*. There are two alternative splice donor sites. Intron variants result in the occurrence of an alternative stop codon and a change of the encoded amino acid C-terminus. The intron of splicing variant 2 ranges from bp 1,329-1,473 and results in an alternative stop codon in the transcript, directing to an alternative C-terminal amino acid composition (→PIGGMPL). The intron of splicing variant 1 ranges from bp 1,363-1,473 and affects only the 3' UTR, but not the amino acid encoding region, which is consistent with the genomic open reading frame (→PIGGYGVYTWGPK). Splicing variant 2 was the predominant form found in the transcripts (ratio 4 to 1). **B**) Graphical overview of splicing variants of *Engam56_2*. Indicated are the GenBank accession numbers and amino acids at the C-terminus of both variants, as well as the region of the intron. **C**) Graphical overview of splicing of *Engam56_1* shows predominantly a splicing variant, like shown for *Engam56_2* splicing variant 2. The intron ranges from bp 1,368-1,512. No other splicing variant was found.

On the genomic level, *gam56_2*, *gam56_1* and *gam82* orthologous genes in *E. nieschulzi* are situated in a cluster and appear as single exon genes (Figure 2, B2); they encode amino acid sequences with calculated masses of approximately 50 kDa (GAM56_2, GAM56_1) and 59.2 kDa (GAM82). The cDNA analysis showed that splicing occurs in the transcripts of the *Engam56* genes (Figure 3). Transcripts of *gam56_2* were always observed as spliced in two alternative versions (Figure 3A, B). The most common one is an intron between 1,328 bp-1,474 bp of the primary transcript, resulting in an alternative stop codon and an altered C-terminal amino acid sequence (splicing variant 2). In the alternative splicing version of *Engam56_2*, a splicing donor after the stop codon was observed, followed by an intron between bp 1,362 and 1,474 (splicing variant 1). The protein encoding part of the gene is not affected by this splicing event (Figure 3A, B). The *Engam56_1* gene is spliced in most of the analysed clones, similar to the *gam56_2* splicing variant 2, but it also occurred in an unspliced version (Figure 3C).

**Figure 4:**
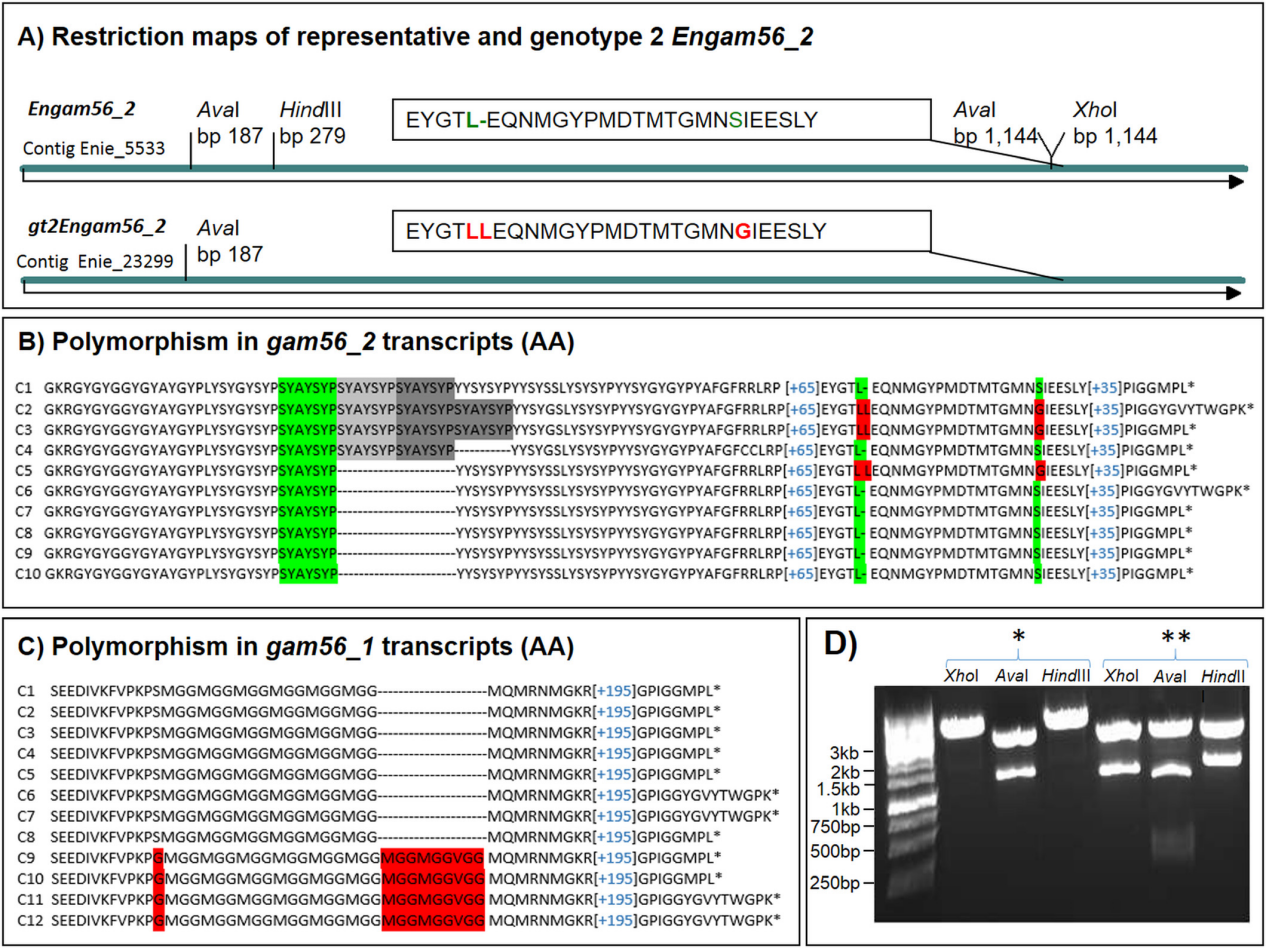
The *gt2Engam56_2* (Enie_23299) and polymorphisms within GAM56 proteins. A) The major differences for identification of the *gt2Engam56_2* (partially encoded by contig Enie_23299 and complete sequenced after PCR amplification) and the representative *Engam56_2* (GenBank AJG00896.1) are different restriction sites (shown in D) and few amino acid changes (AA) (see A) LL(15 AA)G) vs. L(15 AA)S). For full DNA and translated sequences (AA) of the gt2EnGAM56_2, see SF1A and B. B) Sequence analysis of *Engam56_2* cDNA clones (translated sequence is shown) revealed a polymorph region (SYAYSYP motif) in some clones (C1-C4), which is present in both *Engam56_2* versions. Both versions are also alternatively spliced. C) Sequence analysis of *Engam56_1* cDNA clones (translated sequence is shown) revealed two versions (red background, see also SF1D and E). C1-C8 belongs to the high-coverage *gam* locus assembly, whereas C9-C12 is associated with the low-coverage contig Enie_23088, which maps to contig Enie_5532. Please note that both versions were found spliced and unspliced. D) The representative *Engam56_2* and *gt2Engam56_2* can be amplified by the same primer pair, but can be discriminated by restriction site analysis in plasmid DNA. The representative *Engam56_2* (**) has two *Ava*I sites and harbours a *Hind*III and *Xho*I site, the *gt2Engam56_2* (*) has only one *Ava*I site and no *Hind*III or *Xho*I site resulting in different fragment lengths in the agarose gel. The cloning vector pJET1.2 also contains these restriction sites at insert flanking positions.

The *gam56* version fragment encoded by contig Enie_23299 (a low-coverage contig) was detected by analysing cloned *gam56_2*-PCR products via *Hind*III restriction, which is present in the encoding regions of contig Enie_5533 but not in contig Enie_23299. One in twenty analysed clones lacked the *Hind*III restriction and was thus sequenced. The major difference on the amino acid level is a duplicated leucine (Indel) in combination with an alteration from serine to glycine, 17 amino acids downstream (Figure 4A). Comparing the coverage, the contig Enie_23299 has coverage of ca. 14x, whereas Enie_5533 and Enie_5532 have a sequence coverage of ca. 80x and 88x, respectively. We discovered *Engam56* (represented by contig Enie_23299) is an underrepresented gene version, and further designated it as *gt2Engam56_2* (Figure 4A, SF1A). Full alignment of representative EnGAM56_2 and gt2EnGAM56_2 is shown in Supplementary File SF1B.

The *gt2Engam56_2* was spliced, like the representative *Engam56_2* gene. The *gt2Engam56_2* displayed identity of 1,331 bp from 1,362 bp (1,331/1,362), which is 97.8% compatible with the representative *Engam56_2* in the pairwise alignment of DNA sequences, and identity (I) of 95.8% (434/453) and similarity (S) of 98.0% (444/453), respectively in the translated sequences (EMBOSS water). A comparison with the partial sequence of *E. falciformis* GAM56_2 homolog (140 amino acids; Toxodb.org, EfaB_PLUS_28918.g2250.t1) showed that the distance between EfalGAM56_2 and both versions of EnGAM56_2 is higher than the distance between both EnGAM56_2 versions. However, EfalGAM56_2 is more similar to gt2EnGAM56_2 than to the representative EnGAM56_2 (see SF1C).

The alignment of different cDNA clone sequences of *Engam56_2* also revealed the existence of polymorph regions within the genes. In most of the analysed clones, a single SYAYSYP motif is found in the translated amino acid sequence (Figure 4B). Some clones even exhibited this motif triplicated or quadruplicated (Figure 4B). Such differences were also found in amplicons of genomic DNA (data not shown). This occurred in the representative EnGAM56_2, as well as in the gt2EnGAM56_2 version and was present in both splicing variants.

The cDNAs of *Engam56_1* displayed two versions (Figure 4C). The predominant version, also represented by genomic contig Enie_5532 (high coverage), has a sixfold MGG motif ([MGG]_6_). The second version (SF1D, E) encodes an eightfold MGG motif followed by VGG and combined by a glycine instead a serine N-terminal of the first MGG (→S[MGG]_6_ vs. G(MGG]_8_VGG), see Figure 4C). The differences in both motifs are associated with further amino acid alterations in the N-terminal regions (SF1E). The low-coverage contig Enie_23088 was found in the genome data encoding partially this gene version of *Engam56_1*, named *gt2Engam56_1*.

**Figure 5:**
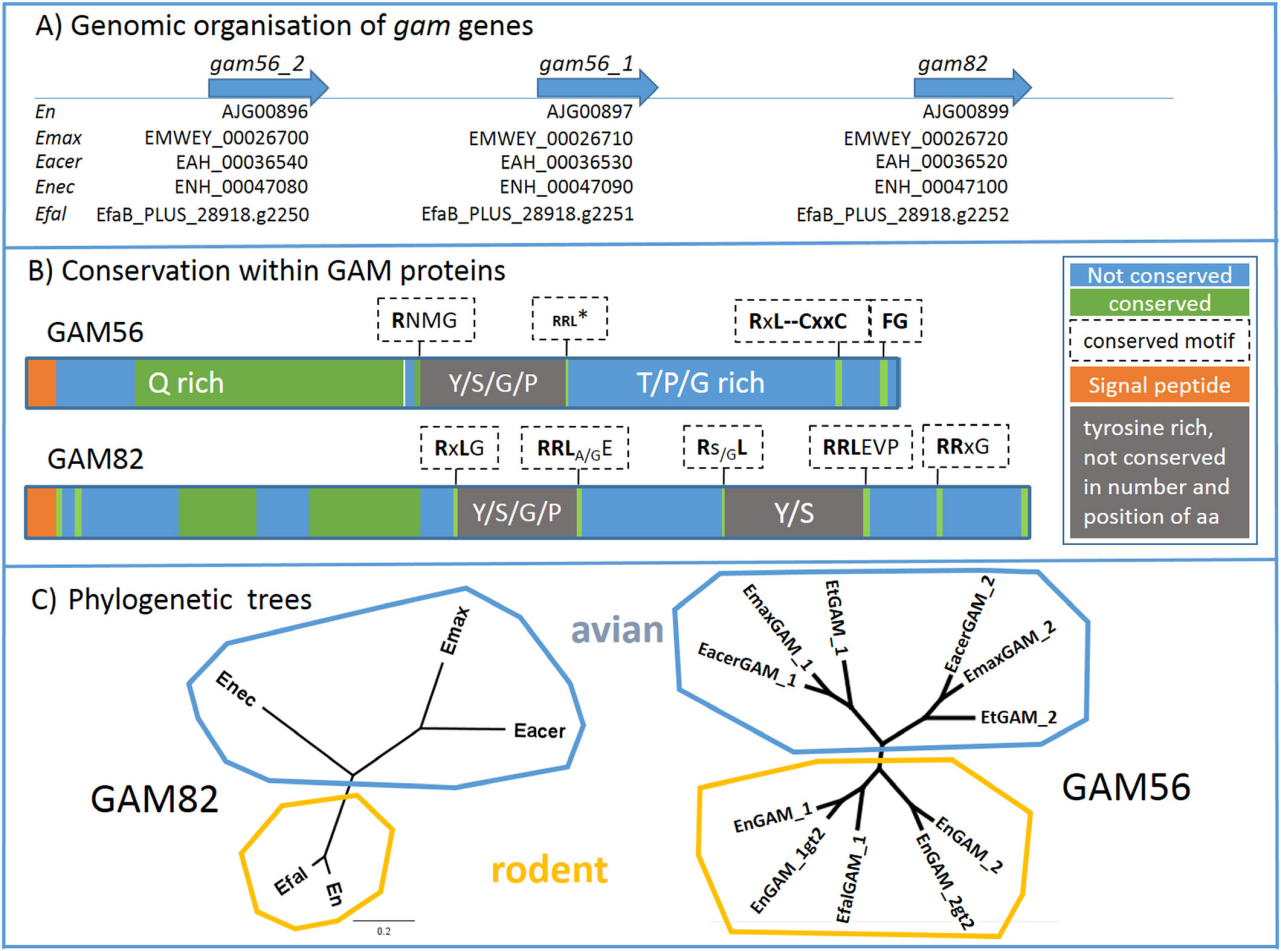
Interspecific analysis of *gam* genes and GAM proteins A) Clustered organisation of *gam56* and *gam82* orthologues. The *gam* genes are situated in a cluster in all investigated *Eimeria* species [GenBank ID: En (*E. nieschulzi*), ToxoDB-ID: Emax (*E. maxima*), Eacer (*E. acervulina*), Enec (*E. necatrix*), Efal (*E. falciformis*)]. B) Conservation within GAM proteins. The consensus structure of GAM56 and GAM82 proteins from different *Eimeria* species are based on the ClustalOmega alignment. RRL*: RRL motif is not conserved in Emax and Eacer GAM56_2, but in all other species and all GAM56_1 variants. (Complete alignment of GAM56 sequences in the SF1G, and H for the GAM82 alignment). C) Phylogenetic trees show distribution of GAM82 and GAM56 in particular branches of rodent and avian *Eimeria* species. GAM82 shows larger distances in the avian branch than in the rodent branch, which confirms the close relationship of *E. falciformis* and *E. nieschulzi.* GAM56 branches within the avian *Eimeria* into two groups, GAM56_1 and GAM56_2, indicating that the common ancestor of this *Eimeria* species already had two GAM56 versions that evolved further in the particular *Eimeria* species.

The visualisation of the genomic situation of *gam* genes in different *Eimeria* species (toxodb.org entries) reveals a similar arrangement of these genes in all investigated species. In all species, we found two consecutive *gam56* genes followed by a *gam82* gene (Figure 5A). Not all sequences were found in their entirety in all species, predominantly due to sequence gaps, and could not be considered in the subsequent alignment to figure out the shared feature of the GAM proteins. For example, *E. tenella* *gam56* genes are situated in a cluster in contig HG675628 (ToxoDB.org), but the *gam82* sequence is completely missing from ToxoDB.org. In *Eimeria* genome assemblies provided by the Sanger Institute, the *Etgam82* is present, but only partially encoded by the contig pathogen_EIMER_contig_00031121 (assembly_2007_05_08).

### The consensus structure of GAM proteins

The identified *gam* gene sequences and those from the literature found in the toxodb.org database were compared using the ClustalOmega alignment tool to examine conserved or non-conserved regions within the encoded amino acids. We found the N-terminal amino acids encoding a signal peptide (SignalP 4.0). In GAM56s, one tyrosine-rich domain and in GAM82 two tyrosine-rich domains are visible (Figure 5B).

In the GAM56 proteins, a less conserved region was subsequent to the signal peptide, flanked by a longer conserved glutamine-rich region. After a short, less conserved area, a conserved motif (RNMG) occurs, flanking the tyrosine-rich region. The tyrosine-rich domain itself is not conserved regarding number or position of amino acids. The domain contains tyrosine alternating with serine, glycine, or proline and C-terminal flanked by an RRL motif followed by a CxxC motif in 4-10 amino acids distance. The C-terminus is of species-dependent length and not conserved. Only an accumulation of proline, glycine and threonine is found. Conserved motifs are only an RxL and FG motif (see Figure 5B and SF1G for alignments and sequence details).

In the GAM82 protein, non-conserved and conserved regions alternate until the first tyrosine-rich domain begins. In this domain, only few tyrosines are conserved in their position, the majority of tyrosines alternate with serine, glycine and prolines at different positions. These four amino acids are frequent in this region, without conservation of the numbers and position, but the recognised conserved limiting protein motifs RxLG and RRL_A/G_E flank the tyrosine-rich domain. Continuing further in the C-terminal direction, another non-conserved domain occurs, which is trailed by the second tyrosine- and serine-rich region. Compared to the previous tyrosine-rich domain, less glycine and proline is present. The low number of positionally conserved amino acids is similar in both tyrosine-rich domains. The conserved amino acid motifs confining the second tyrosine-rich domain are Rs_/G_L and RRLEVP. The C-terminus is less conserved in comparison, but rich in threonine and glutamine and harbours a conserved motif RRxG (Figure 5B and SF1H).

The phylogenetic trees of GAM56 and GAM82 clearly indicate the close relationship of *E. nieschulzi* with *E. falciformis* in a rodent lineage (Figure 5C). GAM56 proteins in the avian lineage branch into a GAM56_1 and a GAM56_2 lineage, indicating that the common ancestor of all investigated avian *Eimeria* species already endured two versions of GAM56. In the rodent lineage, this seems to be similar. From *E. falciformis,* only the GAM56_1 is completely available in the genome data, but not GAM56_2 because of long sequence gaps. This might have the same reason as in the *E. nieschulzi* genome, where the short Illumina sequence reads of duplicated, but identical sequences were not correctly considered in the genome assembly.

## Discussion

GAM proteins are common in avian *Eimeria* species and have been characterised in several studies [3–6,8,16,18–19,28,46]. The identification of these proteins in *E. nieschulzi* aimed to find out the common feature of the GAM proteins within the genus. In the past, only avian *Eimeria* species were examined. Rodent *Eimeria* species are thought to be a good model outside this group. Sequence data were the basis of the study. A cDNA library analysis provided information about *Engam82* and data were confirmed by the subsequent genome project.

Macrogametocytes of *E. nieschulzi* were isolated and concentrated (modified after [32,34]) (Figure 1). The pre-treatment of the tissue and the digestion of host cells, followed by the release of gamonts, showed considerable differences to purification protocols of other avian *Eimeria* species [31–32,34,46]. The extracellular matrix of the rat small intestine was treated with proteolytic enzymes (collagenase, hyaluronidase, trypsine) (data not shown). In addition to the tissue disintegration and cell separation, these enzymes led to a loss of gamont integrity (data not shown). The preparation of a cDNA library from purified *E. nieschulzi* gamonts and the following sequence analysis showed low contamination levels with host cDNA.

Genomic contigs with partial *Engam56* gene sequences were identified in the genome data. Three contigs encoded a 5' end of a *gam56* gene (Figure 2A) and one contig encoded a 3' end (Figure 2A). By PCR and RACE-PCR, we were able to obtain the remaining sequence data and discovered that the 3' ends of all *gam56* variants were nearly identical. This can be explained by the genome sequencing method used, which produced only short reads. For the whole genome, a 100x sequence coverage was planned (estimated genome size: 60 megabases). Since the contig coverage for two 5' contigs was 79.9x and 88.41x, respectively and the 3' contig had coverage of 163.79x, we assumed that the 3' contig obviously included the reads of all *gam56* variants. After reconstruction of the whole *gam* locus of *E. nieschulzi,* we found a clustered organisation of *gam* genes, like in other *Eimeria* species (Figure 5A).

The third identified *gam56* gene 5' contig had coverage of only 14.37x (Figure 2A) and represented a sequence homolog to a part of contig Enie_5533. In amplified PCR products, this contig Enie_23299 gene version was also underrepresented (ratio 1:10), which explains the low coverage in the genome data. It encodes *gam56_2* of a second genotype (gt2) of *E. nieschulzi* and can be discriminated from representative *gam56_2* version by restriction enzyme analysis and by few altered amino acids in the C-terminus of the translated sequence (Figure 4, SF1B). The occurring InDel (encoding leucine-leucine) in *gt2Engam56_2* appears to be a reliable marker for the recognition of the second genotype. The *Engam56_1* gene was also found in two versions, each with a different number of encoded MGG repeats and further amino acid changes (Figure 4, SF1D and E). The *Engam56_1* encoding the sixfold MGG-motif is the representative version encoded by contigs Enie_5532 and Enie_10086. The eightfold MGG is present in the *Engam56_1* of the second genotype (*gt2Engam56_1)* and is also partially encoded by a low coverage contig.

Further analysis of the *E. nieschulzi* genome data revealed that most of the coverage < 40 contigs had a hit in the contigs with coverage > 40 (performed by Emanuel Heitlinger, personal communication). This indicates that the genome data also includes data from a somewhat genetically distinct, underrepresented subpopulation of *E. nieschulzi*.

However, the occurrence of further genotypes of a species is not unusual and has already been found in avian *Eimeria* [12]. Cryptic, so-called Operational Taxonomic Units (OTU) have been described in *Eimeria* recovered from chickens in Australia [11]. For *Eimeria nieschulzi,* no different strains or subspecies have been described to date.

The distribution of reads to distinct contigs with 10x coverage on the one hand and ca. 90x, on the other, suggests that the two genotypes have some differences between their prepatent periods or reproduction rates, or no cross-over whatsoever.

The *E. nieschulzi* strain used in this study has regularly been passaged in our lab since 2003 and was imported to Europe in the 1990s [2]. A literature search showed that the strain used in our study was the isolated strain isolated and described by Landers (1960) [25], which has probably never been cloned via single oocyst infections [30]. The second *E. nieschulzi* genotype (gt2) was either already existent in the original isolate, introduced by contamination, or reflects a speciation process. Since 2012, we have isolated oocysts directly from the intestine at 168-174 h p.i. Before 2012, they were isolated from the faeces, collected from 7-10 days p.i. This might also have an effect on the parasite populations. A hypothetical selection of the majority of a subpopulation by shortening of the excretion time will positively select the subpopulation with the shorter prepatent period in further passages, whereas parasites with a longer prepatent period will be reduced. Another scenario would be a decreased reproduction rate of genotype gt2 compared to the representative genotype. Potential biological and morphological differences between the genotypes could be found prospectively in further studies by single sporocyst infections and analysis of cloned subpopulations. In the present study, we described its occurrence and potential sequence markers for its discrimination. The focus of this study was the description of *gam* genes in *E. nieschulzi*, and we wanted to give an explanation why the *Eimeria nieschulzi* genome data harbour low- and high-coverage contigs.

Analysis of *gam56* cDNA revealed further interesting details about the *gam* genes. *Eimeria* *gam56* genes were to date described as intronless, single exon genes [22], but were found alternatively spliced in *E. nieschulzi* (Figure 3) in this study. The *Engam56_2* had been found spliced in all investigated RACE-PCR clones from cDNA, whereas *Engam56_1* was also found unspliced (Figure 3). The splicing also occurred identically in the second genotype gene versions. This is the first description of splicing in *gam* genes and we can only speculate about the function. Splicing in *Eimeria* and other Apicomplexa is common [20,24,42]. Particularly, only the C-terminal ends of the GAM56 proteins or the 3' UTRs, respectively, are affected by splicing. Splicing of *Engam* genes might have a regulatory function in GAM protein expression but probably not a structural consequence, because only the C-terminal end of the GAM56 proteins or the 3' UTRs of the cDNA are affected by splicing.The alignment of *Engam56_2* cDNAs revealed an additional tandem repeated motif (Figure 4) in few cDNA sequences independently from the genotype. We can only speculate about the emergence. In *E. tenella* and *E. necatrix* GAM56_2 long, partly degenerated repeats also occur in the protein, differentiating this region from the GAM56_2 of other avian *Eimeria* species. *E. tenella* and *E. necatrix* are closely related species [1] and we see that the repeats in the GAM56_2 proteins of both species are different in length and grade of degeneration (SF1F). Considering this observation, we can debate whether the occurrence of such repeats may already reflect separation processes within a population.

Other proteins, described in *Eimeria* wall formation processes like GAM22 [22,27], EtSWP1 [45] and GAM230 [17] are also encoded in the *E. nieschulzi* genome (data not shown), and support our finding that the repertoire of wall-forming proteins is quite conserved in the genus *Eimeria*. The focus of this study was the comparison of GAM56 and GAM82 proteins within the genus. We found that the general similarity concerning motif order is similar, in contrast to the amino acid composition itself, which is not compellingly conserved over large distances in the proteins (see Figure 5B and SF1). However, conserved regions are also found and conserved motifs flank the tyrosine-rich regions. All motifs (RNMG, RxL, RRL, RRxG) start with an asparagine. An RxL or RNMG motif, respectively were found flanking tyrosine-rich motifs N-terminal and an RRL motif flanks C-terminal (Figure 5B). The N-terminal signal might be involved in processing of GAM proteins into smaller peptides, because they flank the sequences of the smaller GAM peptides characterised by Belli et al. (2003) [5]. The function of the RRL motif flanking the tyrosine-rich region at the C-terminus remains unidentified, but RRL motifs have been described in the *Eimeria* T4A antigen, which becomes proteolytic processed at this site in two fragments and is connected via a disulphide bond between cysteine residues in both fragments [9]. In the *Eimeria* GAM56, two cysteine residues are conserved at a distance of 4-10 amino acids C-terminal to the RRL motif, but not N-terminal or in the GAM82. We assume that the RRL motif is involved in processing of GAM56 and GAM82, because Mouafo et al. (2002) described processing of *E. tenella* GAM56 in three fragments [32]. However, Belli et al. (2003) described processing only at one site in *E. maxima* GAM56, and two in the GAM82, respectively [5]. ProP 1.0 Server also predicts cleavage sites at the conserved motifs and supports our hypothesis. Further analysis of peptide sequences could shed more light on the processing sites of *Eimeria* GAM proteins.

In conclusion, we analysed the genome of *E. nieschulzi* concerning the *gam56* and *gam82* orthologous genes. We found, like in avian *Eimeria* [7–8,22,32], two *gam56* orthologous genes and one *gam82* orthologous gene, all arranged in a cluster as in other *Eimeria* species, but these genes encode proteins with a lower mass than avian *Eimeria* GAM proteins. RACE-PCR and cDNA clone analysis enabled us to determine the 3' ends of the *gam* gene transcripts and we observed that *gam56* genes were found mainly alternatively spliced.

This study is the first detailed analysis of rodent *Eimeria* *gam* genes and the first description of alternative splicing of these genes in the genus *Eimeria.* Additionally, we showed that the *E. nieschulzi* genome data also include sequence data of a closely related but distinct second genotype of *E. nieschulzi,* indicated by low-coverage contigs and mirrored in slightly different *gam56* versions. Further sequence analyses will probably uncover further subpopulations or species and depict the real diversity of coccidian parasites, which is so far mainly characterised by oocyst morphology and host specificity.

## Conflict of interest

The authors declare that they have no conflicts of interest in relation to this article.

## Supplementary Files

SF1
A) DNA sequence of *gt2Engam56_2*;
B) Sequence alignment of representative EnGAM56_2 and variant version gt2EnGAM56_2;
C) Alignment of 140 amino acids (translated DNA) of EfalGAM56_2 and EnGAM56_2;
D) DNA sequence of *gt2Engam56_1*;
E) Sequence alignment of representative EnGAM56_1 and variant version gt2EnGAM56_1;
F) Comparison of different repeat length in GAM56_2 homologs in two closely related avian *Eimeria* species;
G) Sequence alignment of GAM56 proteins;
H) Sequence alignment of GAM82 proteins.
SF 2: Obtaining *Engam82* sequence from cDNA library;
SF 3: Contigs_cov_below_40;
SF 4: Contigs_cov_above_40. The Supplementary Material is available at http://www.parasite-journal.org/10.1051/parasite/2017049/olm.
