# Supplementary material for: Identification and analysis of Eimeria nieschulzi gametocyte genes reveal splicing events of gam genes and conserved motifs in the wall-forming proteins within the genus Eimeria (Coccidia, Apicomplexa)
Source: Parasite. 2017 Dec 6;24:50. doi: 10.1051/parasite/2017049 (PMC5718062; doi:10.1051/parasite/2017049)
Supplement: Supplementary file 1 — SF1 A) DNA sequence of gt2Engam56_2; B) Sequence alignment of representative EnGAM56_2 and variant version gt2EnGAM56_2; C) Alignment of 140 amino acids (translated DNA) of EfalGAM56_2 and EnGAM56_2; D) DNA sequence of gt2Engam56_1; E) Sequence alignment of representative EnGAM56_1 and variant version gt2EnGAM56_1; F) Comparison of different repeat length in GAM56_2 homologs in two closely related avian Eimeria species; G) Sequence alignment of GAM56 proteins; H) Sequence alignment of GAM82 proteins. SF 2: Obtaining Engam82 sequence from cDNA library; SF 3: Contigs_cov_below_40; SF 4: Contigs_cov_above_40. [file parasite-24-50-s1.zip › parasite170085-1-olm/SF 1rev.docx]

**A) DNA sequence of *gt2Engam56_2***

**1 ATGACTCGCC TCAGCCTGTG CGCCCTGACT GTGGCTCTTG CCGCAGGGCA**

**51 CTGCTTGGCC GAGCCTAGTA CAGTTGAACG CGCTATGCAC CGTTTTGAAG**

**101 AGATGGAGGT TTTTGACGAC GCCGAGAGCA CCACCACCAC TACCACTACT**

**151 ACCACCACCA AGACAACGAC TCCCATAATT GAGGCCCCCG AGAAGTTCTT**

**201 GGAAAACTTT ATCAAGTCCC TGCAGAGACA GCTGCTACTC CAGGAAACTC**

**251 TTATGAAACA ACTTATGAAT GATATTTTAA ACTATCTGAG GGACATCGCA**

**301 AAGAACTATA CTCTGGGAGA CACACAGCCC TCCGCCATTA CAAAGGTCAA**

**351 TGAGATGATG GAGATGCTCA GCACCCGTAT GGCCACCGCC CTCCAGGGTG**

**401 TTGATGAACT GATGATGAGC AGCGATAGTC TTGACGGCGA GACTTTGCGC**

**451 AGCGTAACCA CGAAATTCAT GAAAGAGGTG CGTGTTCAGG ACATCGTAGT**

**501 CGACGCTCTG TTCTCCTCCC TCCGCGGCAC ACAGACCAAC GCCTTTCTCA**

**551 CTGGCGCTAC CGCCGCCCAA GAAAAGGACG CATACACCGC TGCCAACCGC**

**601 GCTGCCGAGG AGTTCCTGTC CCGCATGTAC CATAACCTGC GTGTCGCTGG**

**651 TATCTCGGAG GAGGACATCG TAAAATTCGT ACCCAAGCCT GGCATGGAGG**

**701 GCATGCAGAT GCGGAACATG GGAAAGAGGG GCTATGGATA CGGTGGATAC**

**751 GGTTATGCTT ATGGCTATCC TTTGTACAGC TATGGTTACA GCTATCCTTC**

**801 CTATGCTTAC AGCTATCCTT ACTATTCATA CAGCTATCCT TACTACAGCT**

**851 ACAGCTCGCT CTACAGCTAC AGCTATCCTT ACTACAGCTA CGGCTATGGC**

**901 TACCCGTACG CTTTCGGCTT CCGTCGTCTT CGTCCCAACT CCTGCCCCGG**

**951 ATGCCCCCCC GGCCCCCCCG TCCCTGTCAC TGGTGTGTCT GAGGTGCCAA**

**1001 TGGGTGTCCC CCCGCAGAAA CCAGTTGTGC CTCCTTTCCG CTCCATGGGG**

**1051 GAGGAGACTC TGGGCATGGG ATCCCCCTCC CCTATGGGTA TGGGATACAC**

**1101 GGACCCCATG ATGGGCTATG GCATGGGCAG TGAGTACGGA ACTCTTCTTG**

**1151 AGCAGAACAT GGGCTACCCT ATGGATACCA TGACCGGGAT GAACGGTATC**

**1201 GAAGAGAGCC TCTACAACAC CTATGGCGGC ATGCCCGCTG GCTACCGCAA**

**1251 CCTTGCTCCC ATGGAGTTTC CTGGCCTCTT CCCTGAGTCT GGCATGCCCA**

**1301 CCGCCCCTTT CGGCTTTGGC CCTATTGGCG GGTATGGTGT ATACACCTGG**

**1351 GGTCCTAAAT AA**

**B) Sequence alignment of representative EnGAM56_2 and variant version gt2EnGAM56_2**

CLUSTAL O(1.2.4) multiple sequence alignment of EnGAM56_2 variants

represent.EnGAM56_2 MTRLSLCALTVALAAGHCLAEPSTVERAMHRFEEMEAFDDAERTTTTTTTTTTRTTTPLI

gt2EnGAM56_2 MTRLSLCALTVALAAGHCLAEPSTVERAMHRFEEMEVFDDAESTTTTTTTTTTKTTTPII

************************************.***** **********:****:*

represent.EnGAM56_2 EAPEKFLENLLKSLQKQLLLQETLMRQLMNDILSFLRDIAKNFALGDTQPPAITKVNEMM

gt2EnGAM56_2 EAPEKFLENFIKSLQRQLLLQETLMKQLMNDILNYLRDIAKNYTLGDTQPSAITKVNEMM

*********::****:*********:*******.:*******::******-*********

represent.EnGAM56_2 EMLSTRMATALQGVDELMMSSGSLDGETLRSVTTKFMKEVRVQDIVVDALFSSLRGTQTN

gt2EnGAM56_2 EMLSTRMATALQGVDELMMSSDSLDGETLRSVTTKFMKEVRVQDIVVDALFSSLRGTQTN

*********************.**************************************

represent.EnGAM56_2 AFLTGATAAQEKDAYAAANRAAEEFLSRMYHNLRVAGISEEDIVKFVPKPGMEGMQMRNM

gt2EnGAM56_2 AFLTGATAAQEKDAYTAANRAAEEFLSRMYHNLRVAGISEEDIVKFVPKPGVEGMQMRNM

************************************************************

represent.EnGAM56_2 GKRGYGYGGYGYAYGYPLYSYGYSYPSYAYSYPYYSYSYPYYSYSSLYSYSYPYYSYGYG

gt2EnGAM56_2 GKRGYGYGGYGYAYGYPLYSYGYSYPSYAYSYPYYSYSYPYYSYSSLYSYSYPYYSYGYG

************************************************************

represent.EnGAM56_2 YPYAFGFRRLRPNSCPGCPPGPPVPVTGVSEVPMGVPPQKPVVPPFRSMGEETLGMGSPS

gt2EnGAM56_2 YPYAFGFRRLRPNSCPGCPPGPPVPVTGVSEVPMGVPPQKPVVPPFRSMGEETLGMGSPS

************************************************************

represent.EnGAM56_2 PMGMGYTDPMMGYGMGSEYGTL-EQNMGYPMDTMTGMNSIEESLYNTYGGMPAGYRNLAP

gt2EnGAM56_2 PMGMGYTDPMMGYGMGSEYGTLLEQNMGYPMDTMTGMNGIEESLYNTYGGMPAGYRNLAP

********************** ***************.*********************

represent.EnGAM56_2 MEFPGVFPESGMPTAPFGFGPIGGYGVYTWGPK*

gt2EnGAM56_2 MEFPGVFPESGMPTAPFGFGPIGGYGVYTWGPK*

****************************************

Differences in amino acid composition are marked with red background. Single amino acid polymorphisms were found in the representative EnGAM56_2 (grey background). The part of gt2EnGAM56_2, which is represented in contig Enie_23299, is highlighted in blue.

**C) Alignment of 140 amino acids (translated DNA) of EfalGAM56_2 and EnGAM56_2**

repEnGAM56_2 MTRLSLCALTVALAAGHCLAEPSTVERAMHRFEEMEAFDDAERTTTTTTTTTTRTTTPLI

EfalGAM56_2 MTRLSLCALTVALAAGHCLAEPSTVERAMHRFEEMEVFDDAESTTTTTTTTTTTTTTPAV

gt2EnGAM56_2 MTRLSLCALTVALAAGHCLAEPSTVERAMHRFEEMEVFDDAESTTTTTTTTTTKTTTPII

************************************.***** ********** **** :

repEnGAM56_2 EAPEKFLENLLKSLQKQLLLQETLMRQLMNDILSFLRDIAKNFALGDTQPPAITKVNEMM

EfalGAM56_2 ETPENFLENFIKSLQRQLQLQENLMRQLMDDIVNFLKDISKNFTLGDTQSSAITKVNEMI

gt2EnGAM56_2 EAPEKFLENFIKSLQRQLLLQETLMKQLMNDILNYLRDIAKNYTLGDTQPSAITKVNEMM

*:**:****::****:** ***.**:***:**:.:*:**:**::***** ********:

repEnGAM56_2 EMLSTRMATALQGVDELMMS

EfalGAM56_2 EMLSTRMATALQGVDELMMS

gt2EnGAM56_2 EMLSTRMATALQGVDELMMS

********************

**Results of pairwise alignment:**

EnGAM56_2 vs. gt2EnGAM56_2 I/S 93.7%/97.1%

gt2EnGAM56_2 vs. EfalGAM56_2 I/S 91.2%/95.4%

EnGAM56_2 vs. EfalGAM56_2 I/S 89.2%/93.3%

**D) DNA sequence of *gt2Engam56_1***

**1 ATGGTTCGTC TTATCCTTTC CGCCATCACT GTGGCTGTTG CCGCAGGGCG**

**51 TTGCATGTCC GAGCCCATCG GCGAGCCTGA AATCCAAACC TACGAGGCAA**

**101 TGGGGGGCAA TAACCAGCAG GAACAGGTTA ATACCTACGT GAACACAATG**

**151 GAAGACGCTC CCACTACTCC AACTAAAAGA CCTGGTGCTG AGGAGATGCT**

**201 CGCCCAGCTG CCCAAGCTAG TGGAGAAACA GCTGCAGCTC CAGGATGATC**

**251 TTATGAACAA AATTATTAAG GATGTCGACG AGTACATGAA GGAGGCCAGC**

**301 AAGGCCTTTA CCTTTGAAAA CACCCAGTCC TCCGCCATTA CAAAGGTCAA**

**351 CGACATGATG GAGAAGATCA GCACCCGCAT GGCCACCGCC CTCCAGGGCG**

**401 CCAACGAACT CATGGCCAAC AGCGAAGCTC TGGACACCGA GACGTTGCGC**

**451 AGCGCTACCA TGAAGTTCAT GAAAGAGGTG CGTGTTCAGG ACATCGTAGT**

**501 CGACGCTCTG TGGGCCTCCC TCCGCGGCAC GCAGACCAAC GCCTTTCTCA**

**551 CTGGCACCGC CGGCTCTGAG AAGGATGCTT GCACCGCTTC GAAGCGCGCC**

**601 GAGGAGTTGC TGTCCCGCAT GTACCATAAC CTGCGTGCCG CTGGTATATC**

**651 GGAGGAGGAC ATCGTAAAAT TCGTACCCAA GCCTGGCATG GGTGGCATGG**

**701 GTGGAATGGG TGGCATGGGT GGAATGGGTG GAATGGGTGG CATGGGTGGC**

**751 ATGGGTGGAG TGGGTGGCAT GCAGATGCGG AACATGGGAA AGAGGGGATA**

**801 TGGATACGGT GGATACAGTT ATGGTTATGG CTATCCTTTG TACAGCTATG**

**851 GTTACAGCTA TCCTTCCTAT GCATACAGCT ATCCTTCCTA TGCATACAGC**

**901 TATCCTTACT ACAGCTACGG CTCGCTCTAC AGCTACAGCT ATCCTTACTA**

**951 CAGCTACGGC TATGGCTACC CGTACGCTTT CGGCTTCCGT CGTCTTCGTC**

**1001 CCAACTCCTG CCCCGGATGC CCCCCCGGCC CCCCCGTCCC TGTCACTGGT**

**1051 GTGTCTGAGG TGCCAATGGG TGTCCCCCCG CAGAAACCAG TTGTGCCTCC**

**1101 TTTCCGCTCC ATGGGGGAGG AGACTCTGGG CATGGGATCC CCCTCCCCTA**

**1151 TGGGTATGGG ATACACGGAC CCCATGATGG GCTATGGCAT GGGCAGTGAG**

**1201 TACGGAACTC TCGAGCAGAA CATGGGCTAC CCTATGGATA CCATGACCGG**

**1251 GATGAACAGT ATCGAAGAGA GCCTCTACAA CACCTATGGC GGCATGCCCG**

**1301 CTGGCTACCG CAACCTTGCT CCCATGGAGT TTCCTGGCGT CTTCCCTGAG**

**1351 TCTGGCATGC CCACCGCCAC TTTCGGCTTT GGCCCTATTG GCGGGATGCC**

**1401 CTTGTAA**

**E) Sequence alignment of representative EnGAM56_1 and variant version gt2EnGAM56_1**

CLUSTAL O(1.2.3) multiple sequence alignment of EnGAM56_1 variants

represent. EnGAM56_1 MVRLILSAITVAVAAGRCMSEPIGEPEIQTYEAMGGNNQQEQVNTYVNTMEDAPTTPTKR

gt2EnGAM56_1 MVRLILSAITVAVAAGRCMAEPIGEPEIQTYEAMGGNNQQEQVNTYVNTMEDAPTTPTKR

************************************************************

represent. EnGAM56_1 PGAEEMLAQLPKLVEKQLQLQDDLLNKIIKDVDEYMKEASKAFTFENTQSSAITKVNDMM

gt2EnGAM56_1 PGAEEMLAQLPKLVEKQLQLQDDLMNKIIKDVDEYMKEASKAFTFENTQSSAITKVNDMM

************************:***********************************

represent. EnGAM56_1 EKISSRMATALQGANELMANSEALDTETLRSATMKFMKEVRVQDIVVDALWASLRGTQTN

gt2EnGAM56_1 EKISTRMATALQGANELMANSEALDTETLRSATMKFMKEVRVQDIVVDALWASLRGTQTN

****:*******************************************************

represent. EnGAM56_1 AFLTGTAGSEKDVCTASKRAEELLSRMYHNLRVAGISEEDIVKFVPKPSMGGMGGMGGMG

gt2EnGAM56_1 AFLTGTAGSEKDACTASKRAEELLSRMYHNLRAAGISEEDIVKFVPKPGMGGMGGMGGMG

************.*******************.***************.***********

represent. EnGAM56_1 GMGGMGG---------MQMRNMGKRGYGYGGYGYAYGYPLYSYGYSYPSYAYSYPYYSYS

gt2EnGAM56_1 GMGGMGGMGGMGGVGGMQMRNMGKRGYGYGGYSYGYGYPLYSYGYSYPSYAYSYPSYAYS

******* ****************.*.********************-*:**

represent. EnGAM56_1 YPYYSYGSLYSYSYPYYSYGYGYPYAFGFRRLRPNSCPGCPPGPPVPVTGVSEVPMGVPP

gt2EnGAM56_1 YPYYSYGSLYSYSYPYYSYGYGYPYAFGFRRLRPNSCPGCPPGPPVPVTGVSEVPMGVPP

************************************************************

represent. EnGAM56_1 QKPVVPPFRSMGEETLGMGSPSPMGMGYTDPMMGYGMGSEYGTLEQNMGYPMDTMTGMNS

gt2EnGAM56_1 QKPVVPPFRSMGEETLGMGSPSPMGMGYTDPMMGYGMGSEYGTLEQNMGYPMDTMTGMNS

************************************************************

represent. EnGAM56_1 IEESLYNTYGGMPAGYRNLAPMEFPGVFPESGMPTAPFGFGPIGGMPL

gt2EnGAM56_1 IEESLYNTYGGMPAGYRNLAPMEFPGVFPESGMPTATFGFGPIGGMPL

************************************-***********

Differences in amino acid composition are marked with red background. Single amino acid polymorphisms were found in EnGAM56_1 (grey background). The part of gt2EnGAM56_1, which is encoded by contig Enie_23088, is highlighted in blue.

**F) Comparison of different repeat length in GAM56_2 homologs in two closely related avian *Eimeria* species**


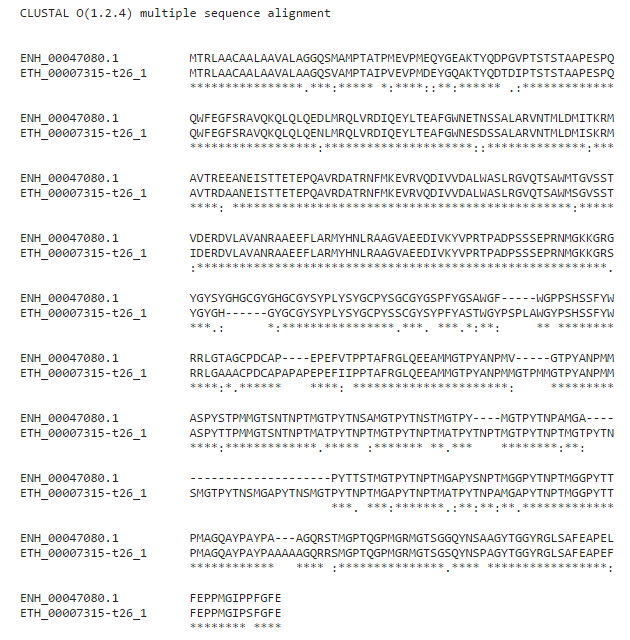


The blue box indicates the repeat region in both species, *E. tenella* and *E. necatrix* GAM56_2 (ETH_00007315-t26_1, syn. EtGAM59, ENH_00047080.1).

**G) Sequence alignment of GAM56 proteins**

CLUSTAL O(1.2.4) multiple sequence alignment

EtGAM56_2 MTRLAACAALAAVALAAGQSVAMPTAIPVEVPM-DEYGQAKTYQDTD-------------

EmaxGAM56_2 MTRFAACV-LAAVLFAAGPSSALPTPI--GDPF-RRLGEMETYQTGGEGPSPNPTPSPPA

EaGAM56_2 MTRLTACA-LAAVALLAGPSSALPTPL--EGTY-HRLGEMETYEPSG---APTPTPTPAA

EnGAM56_2 MTRLSLCA--LTVALAAGHCLAEPSTVERA--M-HRFEEMEAFDDAERTTTTTTTT----

EnGAM56_1 MVRLILSA--ITVAVAAGRCMSEPIGEP-E--I-QTYEAMGGNNQQEQVNTYVNTM----

EtGAM56_1 MTRLSLCA--LAVALAVGQSLAVPTT--VENTVH-PYSEMGTYQEGEAPGAPDESS----

EmaxGAM56_1 MTRLGLAA--VALALAVGPSMAVPSTTPVENQVH-PYSEMSTYQEGSAPGAPEDTT----

EaGAM56_1 MTRLGFCT--LALALAVGPAMAVPSTTSVESTVLPTYTEMGTYQEGGAPGTPDDTT----

*.*: .. :: . .* . : * :

EtGAM56_2 --IPTSTSTAAPESPQQWFEGFSRAVQKQLQLQENLMRQLVRDIQEYLT---EAFGWNES

EmaxGAM56_2 EGSSSSTSTAAPDTAQQWFEGFNRAVQKQLQLQESLMRQLMEDIQQYLS---EALGWNEN

EaGAM56_2 EGSSSSTSTAAPDAAQQWFEGFSRAVQKQLQVQESLMRQLMTDIQEYLT---DALGWNEN

EnGAM56_2 --TTRTTT-PLIEAPEKFLENLLKSLQKQLLLQETLMRQLMNDILSFLRDIAKNFALGDT

EnGAM56_1 --ENAPTTPTKRPGAEEMLAQLPKLVEKQLQLQDDLLNKIIKDVDEYMKEASKAFTFENT

EtGAM56_1 --TTTTTPSPSPEAPDQWLENFVRAVQRQLQLQESMMRQLVKEIQEYLS---RAFNWDEN

EmaxGAM56_1 --TT-TTSSPVSDGAEQWLESFVRAVQRQLQLQDQMMRQLMRDIQEYLS---TAFNWAEN

EaGAM56_1 --TT-TTTSPTSEGGDNWLENFIRAVQRQLQLQENMMRQLIRDIQEYLS---NAFNWAEN

* :: : : : :::** :*: ::.::: :: .:: : :.

EtGAM56_2 DSSALARVNTMLDMISKRMAVTRDAANEI--STTETEPQAVRDATRNFMKEVRVQDIVVD

EmaxGAM56_2 GSSALERVNAMLEMISSRMAITREAATEMAIGSSETEEQAAREATQKFMREVRVQDIVVD

EaGAM56_2 GSSALERVNAMLEMISSRMAITREAATDMV-STAEAEEQAAREASRKVMREVRVQDIVVD

EnGAM56_2 QPPAITKVNEMMEMLSTRMATALQGVDELMMSSGSLDGETLRSVTTKFMKEVRVQDIVVD

EnGAM56_1 QSSAITKVNDMMEKISSRMATALQGANELMANSEALDTETLRSATMKFMKEVRVQDIVVD

EtGAM56_1 QSAAYNRVNEMMDMITNRMTTALDGANELMATSETMDPETLRRATRKYMKEVRVQDVVVD

EmaxGAM56_1 QSTAYTRVTEMMDMISNRMNAAMDSSNELMTTSDTTDPETLRRATRKYMKEVRVQDVLVD

EaGAM56_1 QSTAYTRVTEMMDMISNRMSAAIDSSNELMTASETMDPETLRRTARKYMKEVRVQDVVVD

* :*. *:: ::.** : :. :: : : :: * .: : *:******::**

EtGAM56_2 ALWASLRGVQTSAWMSGVSSTIDERDVLAVANRAAEEFLARMYHNLRAAGVAEEDIVKYV

EmaxGAM56_2 ALWASLRAVQTSTWMSGITGQVEESDVFNAANRAAEEFLVRMYHNLRAAGIAEEDIVKYV

EaGAM56_2 ALWASLRGVQASAWMGGIGGHVEEADVFNAANRAAEEFLARMYHNLRAAGIAEEDIVKYV

EnGAM56_2 ALFSSLRGTQTNAFLTGATA-AQEKDAYAAANRAAEEFLSRMYHNLRVAGISEEDIVKFV

EnGAM56_1 ALWASLRGTQTNAFLTGTAG-SE-KD-VCAASKRAEELLSRMYHNLRVAGISEEDIVKFV

EtGAM56_1 SLWASLRGVQTSAWMSGVTA-VEKEETTPMAGRAAEEFMHRMYHNLRAAGMAEEDITRFM

EmaxGAM56_1 ALWASLRGVQTAAWMNGVTA-IEKEETTPMASRAAEEFLHRMYHNLRAAGMSEEDVAKFI

EaGAM56_1 ALWASLRGVQTAAWMSGVTA-IEKEETTPMASRAAEEFLHRMYHNLRAAGMSEEDVAKFV

:*::***..*: ::: * . : : *.: ***:: *******.**::***:.:::

EtGAM56_2 PRTPADP-------------SSSEPRNMGKKGRSYG-YGHGYGCGYSYPLYSYGCPYSSC

EmaxGAM56_2 PKNAIEE-----------TT-GSPTRNMGRKSRGYYHYNYGYNYGYRYPHYSYYYTHPQP

EaGAM56_2 PKTVIEE-----------TISSSPTRNMGSKSRGYSYYN----YGYRYPHYTYGYAHPYP

EnGAM56_2 PKPGM---------------EGMQMRNMGKRGY-G-YG--GYGYAYGYPLYSYGYSYPSY

EnGAM56_1 PKPSMGGMGGMGGMGGMGGMGGMQMRNMGKRGY-G-YG--GYGYAYGYPLYSYGYSYPSY

EtGAM56_1 PKTEYTT-------------PREQTRNMGRKGRYGYGY--S----YGYPLYSYGYSYPSY

EmaxGAM56_1 PRAEYN--------------PSEQSRNMGRKGRSFYYG--GYPSYYNSPYYSYSSYPSYY

EaGAM56_1 PRGEYT----------------QQPRNMGRKGRFGYYG---YSGYYGYPSYYSYPYSSYY

*: **** :. * * *

EtGAM56_2 ------GYSYPFYASTWGYPSP--------LAWGYPS------------------HSSFY

EmaxGAM56_2 ------FYSYPVAA-YPRYMQN--------YSWGYPV------------------YTPSD

EaGAM56_2 -----TFYSYPTVA-YPRYTYN--------YSWGYPM------------------QSSFG

EnGAM56_2 AYSYPY-YSY----------------SYPYYSYSS----LYSYSYPYYSYGYGYPY-AFG

EnGAM56_1 AYSYPY-YSY----------------SYPYYSYGS----LYSYSYPYYSYGYGYPY-AFG

EtGAM56_1 SYSYP-YYSYPSY-SYPLYSYSY---RYPSYSYSYPLYSYSSYSYPYYSYSYPYYSSSWY

EmaxGAM56_1 NYSYPS-------YSYSSYPSYYRYSSYPYYNYSYPSYYN-YGSYPYYSY---SSYPSWY

EaGAM56_1 SYSYPSYYSYPSYYRYSSYPSYYGYSSYPYYSYGYPSYS--------------YGYPSYY

:.

EtGAM56_2 WRRL------GAAACPDCAPAPAPEPEFII----------PPTAFRGLQEEAMMGTPYAN

EmaxGAM56_2 WGSAPC----FSSSCNDCGRGPQP-----E----PLVHEH-EMFHRALGEEDP-------

EaGAM56_2 WSSPRF----FSSSCSTCNECSSRLPLPGE----PLMHEVEPIHHRRLGEEGP-------

EnGAM56_2 FRRLRP------NSCPGCPPGPPVPVTG--------------------VSEVPMGVPPQK

EnGAM56_1 FRRLRP------NSCPGCPPGPPVPVTG--------------------VSEVPMGVPPQK

EtGAM56_1 WRRLRT------ASCPDCPPGVN--MPPTPLSPMNTPLAGTP-----SPTTTPMMPPMMP

EmaxGAM56_1 WRRLRSL---ATATCPDCPPLTTPSMIPTPPPMMNMMNTPPPMANM-MTSM-----MMNT

EaGAM56_1 WRRLRPGTPGTPATCPDCPPMNTPNMPT-PP-PMNMMNNTPPMMNM-MNNIPPMMNMNTP

: :* *

EtGAM56_2 PMMG----------TPMMGTP--YANPMMASPYTTPMMGTSNTNPTMATPYTNPTMGTPY

EmaxGAM56_2 TMMG----------AHGAPSPYVYGTPMMQDAYGTGPA--------M-------------

EaGAM56_2 MMMG----------GV----PFGYNSHMMPTP-----Q--------A-------------

EnGAM56_2 PVVPPFRSMGEETLGMGSPSPMGMGYT---DPM----M----------------------

EnGAM56_1 PVVPPFRSMGEETLGMGSPSPMGMGYT---DPM----M----------------------

EtGAM56_1 PMVPPTRTLGTEPLTMGMGPMPGYGYPPMM------------------------------

EmaxGAM56_1 PMVPPPRTLGTEAMSLGLA-PIGI----TGAPM----T----------------------

EaGAM56_1 PMVPPSRTLGTEAMGLNMGAPLGMNTPTPLPPM----T----------------------

::

EtGAM56_2 TNPTMATPYTNPTMGTPYTNPTMGTPYTNSMGTPYTNSMGAPYTNSMGTPYTNPTMGAPY

EmaxGAM56_2 --------------------------SAQGYNSGY-PNY----------GYYPSSMNPRF

EaGAM56_2 --------------------------YPNNYNSYYNYNY----------NSFPPTMNSRY

EnGAM56_2 -----------------------GYGMGSEYGTL-EQ-----------------------

EnGAM56_1 -----------------------GYGMGSEYGTL-EQ-----------------------

EtGAM56_1 -------------------------DTMPEFPPF-AE-----------------------

EmaxGAM56_1 -----------------------GFGVPPEFGPFGAE-----------------------

EaGAM56_1 -----------------------GFGIPPEFPPFGGE-----------------------

EtGAM56_2 TNPTMATPYTNPAMGAPYTNPTMGGPYTTPMAGQAYPAYPAAAAAGQRRSMGPTQGPMGR

EmaxGAM56_2 NAATTAA-----------------------TGNMNYA-------------QQSPAAPMGR

EaGAM56_2 NTMPFAA-----------------------N-NANYPQQQG--PTSAAAAAAAQQQPLGR

EnGAM56_2 --------------------------------N-----------------MGYPMDTMTG

EnGAM56_1 --------------------------------N-----------------MGYPMDTMTG

EtGAM56_1 --------------------------------G-----------------MGYPTEPINS

EmaxGAM56_1 --------------------------------G-----------------IGLPTDALGS

EaGAM56_1 --------------------------------G-----------------VGYPMDLLGN

. :

EtGAM56_2 MGTSG-SQYNSPAGYTGGY-------RGLSAFEAPEFFEP--------PM-GIPSFGFE-

EmaxGAM56_2 MGSLG-SPYGYTVTPNNRYRPSTFPVRNLGMWEGPE------------PMMEFMPFGAVE

EaGAM56_2 MGSLG-SPYGYAVTPNSGYQQTTF--RNLGVWEGPEMINE--------PFVPFVPFGMVE

EnGAM56_2 MNSIEESLYNTYGGMPAGY-------RNLAPMEFPGVFPESGM--------PTAPFGFGP

EnGAM56_1 MNSIEESLYNTYGGMPAGY-------RNLAPMEFPGVFPESGM--------PTATFGFGP

EtGAM56_1 IPTMNNMDTPFE--NTTNY-------RNLAPIDMPPFFPEAPMRPTPTPTPTPTPFGFGP

EmaxGAM56_1 TP----EMTPFD--PTTPY-------RTLAPMDLP---PIPPPVFPETPMRPPTPFGFGP

EaGAM56_1 MN---IDATPFETSNTTNY-------RNLAPVDLPTPTPIPPPVFGEPPM-PPTPFGFGP

* * *. : * **

EtGAM56_2 ------------

EmaxGAM56_2 P-----------

EaGAM56_2 P-----------

EnGAM56_2 IGGYGVYTWGPK

EnGAM56_1 IGGYGVYTWGPK

EtGAM56_1 VPP---------

EmaxGAM56_1 APVPP-------

EaGAM56_1 VPTPG-------

Sequence alignment of GAM56 proteins of *E. acervulina* (Ea), *E. maxima* (Emax), *E. nieschulzi* (En), and *E. tenella* (Et). Conserved amino acids are marked in red. The signal peptide is highlighted in yellow and the tyrosine-serine-glycine-proline rich domain is marked in dark grey. Conserved motifs (RNMG, RRL, CxxC, RxL, FG) are shaded in blue. (EnGAM56_2 GenBank ID. AJG00896.1; EnGAM56_1 GenBank ID. AJG00897.1; EmaxGAM56_2 ToxoDB ID EMWEY_00026700-t26_1-p1; EmaxGAM56_1 ToxoDB ID EMWEY_00026710-t26_1-p1; EtGAM56_2 (synonym EtGAM59) ToxoDB ID. ETH_00007315-t26_1-p1; EtGAM56_2 ToxoDB.org ID ETH_00007320-t26_1-p1; EaGAM56_2 ToxoDB ID EAH_00036540-t26_1-p1; EaGAM56_1 ToxoDB ID EAH_00036530-t26_1).

**H) Sequence alignment of GAM82 proteins**

CLUSTAL O(1.2.2) multiple sequence alignment

EaGAM82 MTRAAALAGVLALAAAGGSLALPTVLEGTATPTMEWTEETPIDAEVSVEEMGS-------

EmaxGAM82 MTRAAALAGVLALAAAGSSLALPTVLDTTTGTQVEWTETPLDTTEVTMGEMGS-------

EneGAM82 MARAAAIAGFLALAA-GRGVALPTVNT--AAHPVEWEEAATAVPTAAREAAAAAVSLNEA

EnGAM82 MVRAVA-IGFLALAA-GQALALPTLEN--AQQMGEWEEAAVEAQKAAAASVADTISATQ-

EfGAM82 MVRAVAIAGFLALAA-GQALALPTLEN--AQQMGEWEEAAVEAQKAAAASVADTISATQ-

*.**.* *.***** * .:****: : ** * .: .

EaGAM82 --TS----GSSTGVRMMEAASPTPMTPETPQQQPQPPQQQQQQPQPVTMPEAALEAIMQE

EmaxGAM82 --TTSGTTPTSTGVRMMEAETTTPSTPEAPQQQQQMPQPQPQPQQTTPVPEAVLEAIMQE

EneGAM82 ISSSSSSSSSIPRMVEAAS---------------------EPPQQQTAVQEAVLEAIMRE

EnGAM82 ------------GIRMMGT---------------------EESSQQLAVQEAVLDSIMKE

EfGAM82 ------------GIRMMGT---------------------EESSQQLAVQDAVLDSIMKE

: : * : :*.*::**:*

EaGAM82 IQNMFRTSLVMPGWETVDTAAEAVRNIVGRVRDRLTGSVITSAADMEASTMGSRPTTASA

EmaxGAM82 MQNIFRSSL-VPGWDTVGTAADAVRQIVTRVRERLTGPLMMTEMDTGLGRTG--PLSTTG

EneGAM82 MQNIFRNTLTVPGWETMDTAADSVRQIVARVRERLTTGLADTD-----------------

EnGAM82 VQTIFKNTLTVPGWDNMDSAAETVKQIVDRVRERLAAGFTETE-----------------

EfGAM82 VQTIFKNTLTVPGWDNMDSAAETVKQIVDRVRERLAAGFTETE-----------------

:*.:*:.:* :***:.: :**::*::** ***:**: . :

EaGAM82 AAAAAAGPVAALRGVTNDFLREIMVQEAVLETLWAVLRDAQERPWVLEED-SLQVATTQA

EmaxGAM82 ATGATTGPVAALRGVTNDFLREIMIQEAVLETLWAVVRDAQERPWLVNEQEALHAATADA

EneGAM82 TTGLPLTTAAALRGVTTDFLREIMVQEAVIETLWAVLRDAQARPWAASEQQALHVAAAQA

EnGAM82 AGRTTMSTASALRGVTSDFLKEIMVQEAVIETLWAVLRDSQARPWITNEQQAMQTAAQHA

EfGAM82 AGRTTMSTASALRGVTSDFLKEIMVQEAVIETLWAVLRDSQARPWITNEHQAMQTTAQQA

: .:******.***:***:****:******:**:* *** .*. :::.:: .*

EaGAM82 VEGFLVRMHDRLAATGFTEEEITRLLPRQRNCSRRG----VSGLLDNCQDTPPTRSLGKK

EmaxGAM82 VQGFLGRMHDRLRATGFSEEEVMRLLPRSRNGGCTR----TGGLFDQCNDAPPSRLLGKR

EneGAM82 VQGFLERMQDRLRSTGFSEEEIFRLMPRQRSCAREGAPGGPPGLFEACAE---PRNLGKR

EnGAM82 VQGFLLRMHDRLRATGFTEEEIVKMMPRPKACTLDG----PAGEFDTCPE----RNLGKR

EfGAM82 VQGFLLRMHDRLRATGFTEEEIVKMMPRPKTCNPDG----SGGEFDTCPE----RNLGKR

*:*** **:*** :***:***: :::** : * :: * : * ***:

EaGAM82 GYGGGYYGYGYPSYSYYSYPMYSYTYAPPVYMQ--PLA------------YPTYYSYSWG

EmaxGAM82 MYSTGYYGYGYPSYYSYGYSYPAY-------SH--YPV------------SYPYYGYSWG

EneGAM82 GAPS----YGYPLY-YPSYHFP-AFYSYPAFGCSSCSSSCGYYGGCGCG--GPYWS-SWG

EnGAM82 GGYGGYGGYGYGGYGHGGYGYRSYGYSYPFYGYSSYGYGYPYYGGYSY--GYPYHARSYG

EfGAM82 GG------YGYGGYGYGGYGYRSYGYGYPYYGYSSYS--YPYYGGYSYGYGYPYYARSYG

*** * .* * . *:*

EaGAM82 PTYYYSRGYYGKHGHKHG-YYRRLAEGEGSAALPPAAAAAAALRQAAAEALAEMLPPAAP

EmaxGAM82 PSYYYGSGYYGKHGYKYGHYYRRLAEQEPRPVMPPAAATAAANLRAAAAAAAEVPPPPPP

EneGAM82 -------YYR--------GKHRRLGEGEVAEPIPGAPGG---------------PPGGPM

EnGAM82 YGYPYGYGYR--------GFYRRLGE--VATPESSIPQS---------------IPPIPV

EfGAM82 YGYPYSYGYR--------GFYRRLGE--VAAPESSIPQG---------------IPPIPV

* :***.* *

EaGAM82 AAPVE---DIHPWFSPEDAD------TPLRPPN----------RGLGWASPYT-------

EmaxGAM82 AAV-P---PPPPA-AAAGTP------AMMPPPMMGV-EEPVPFRSL---------YPSYS

EneGAM82 GAPMGAPMGAPPLLG------------GPNFEPAGFHLPGEQPRSLFSSSFYPAFYPSYS

EnGAM82 DEQMGFPAPGVPGFGLNGMGMGLGGMGAMGPPVGPLGMDNAAPRSLY-ASFARGFYPSYS

EfGAM82 DEQMGFPAPGVPGFGLNGMGM-----GGMGLPVGPFGNENATPRSLY-TGFARGFSYPY-

* . .

EaGAM82 -------RYYSSYSYYTPY----SFYPRYSYA----P---FSYYSRPLYSYPYTY---SS

EmaxGAM82 WSYPAYTRVSPSYSYYTPSYSSSYYYPRYNYAYNYPL---YSDYSWYDYSYPLAY---SS

EneGAM82 YSY------------SYPYSYSSSSSSSYSFPYSSSSSYSFPYSSSYSYSFPYSSSYSYS

EnGAM82 YGY------------SYPSYIYGYSYPSYSYGYSY---------PSYSY--------GYS

EfGAM82 -----------------------------------------------SY--------GYS

* *

EaGAM82 YPYSYSYSYPYT-YSYSYPYSYSY-----P-SFYRRLEVPDINASTSSQQQQQQQQQEED

EmaxGAM82 YS-----SYPLSYSSYSYPLSYTY-----PSAFYRRLEVPDLTTTTTTHHEQQQQQQQES

EneGAM82 FPYSYSYSYPAFYGAPLYPLYSFPLLRYRAWGPWRRLEVPELSPSQQSTAAPSE------

EnGAM82 YPSYYSYSYPSYSLGYSYPSYYSYGYPFRSFGFFRRLEVPDLSVPSTTVPMDNIT-----

EfGAM82 YPSYYSYAYPSYSYGYSYPSYYSYAYPFRSFGFFRRLEVPDLSVPSTTVPMDSIT-----

: :** . ** . :******::. : .

EaGAM82 TTTTTASLGGRMSISGVQTRSTSTNSLRRTGQRSETL-------------N--TPTYYSN

EmaxGAM82 TTTAVP----TETITTPSTRNTHSSSLRRVGERYEPI-------------TPTQRTFYNN

EneGAM82 ---ASPSTG--------RSTG----LFRRAGVTTGVTSGVTTGVTTGVTSGHTSGQYYNA

EnGAM82 --PNTTSTG--------RTGGFSQNSFRRVGES---------------RTPMTTGSFYTG

EfGAM82 --PNT-STG--------RTGGFTQNSFRRVGEV---------------RTPMTTGSFYTG

: :**.* :*.

EaGAM82 TDTPRNTVYTPEHLEEPQTQTATQTEWETYN-

EmaxGAM82 TEGTNNPVYTPENLTEDE----PQTVWETYN-

EneGAM82 HSQVPAEAAVYSNE-QQQQQQHEQPLWDTSYN

EnGAM82 QNSPL-NIETPFEP-QQAVNENVSATWETTYN

EfGAM82 QNTPLNNIETPFEP-QQAVNENVPSTWETTYH

. . . : *:*

Sequence alignment of GAM82 proteins of *E. acervulina* (Ea), *E. falciformis* (Ef), *E. maxima* (Emax), *E. nieschulzi* (En), and *E. necatrix* (Ene). Conserved amino acids are marked in red. The signal peptide is highlighted in yellow and the tyrosine-serine-glycine-proline rich domain is marked in dark grey. Conserved motifs (RxLG, RRL_A/G_E, R_S/G_L, RRLEVP, RRxG) are shaded in blue. (EaGAM82 ToxoDB ID EAH_00036520-t26_1-p1; EmaxGAM82 ToxoDB ID EMWEY_00026720-t26_1-p1; EneGAM82 ToxoDB ID ENH_00047100.1-p1; EnGAM82 GenBank ID KJ939342.1; EfGAM82 ToxoDB EfaB_Contig_28918 bp 473-1982, translated).
