# Supplementary figures and images for: Identification and analysis of Eimeria nieschulzi gametocyte genes reveal splicing events of gam genes and conserved motifs in the wall-forming proteins within the genus Eimeria (Coccidia, Apicomplexa)
Source: Parasite. 2017 Dec 6;24:50. doi: 10.1051/parasite/2017049 (PMC5718062; doi:10.1051/parasite/2017049)

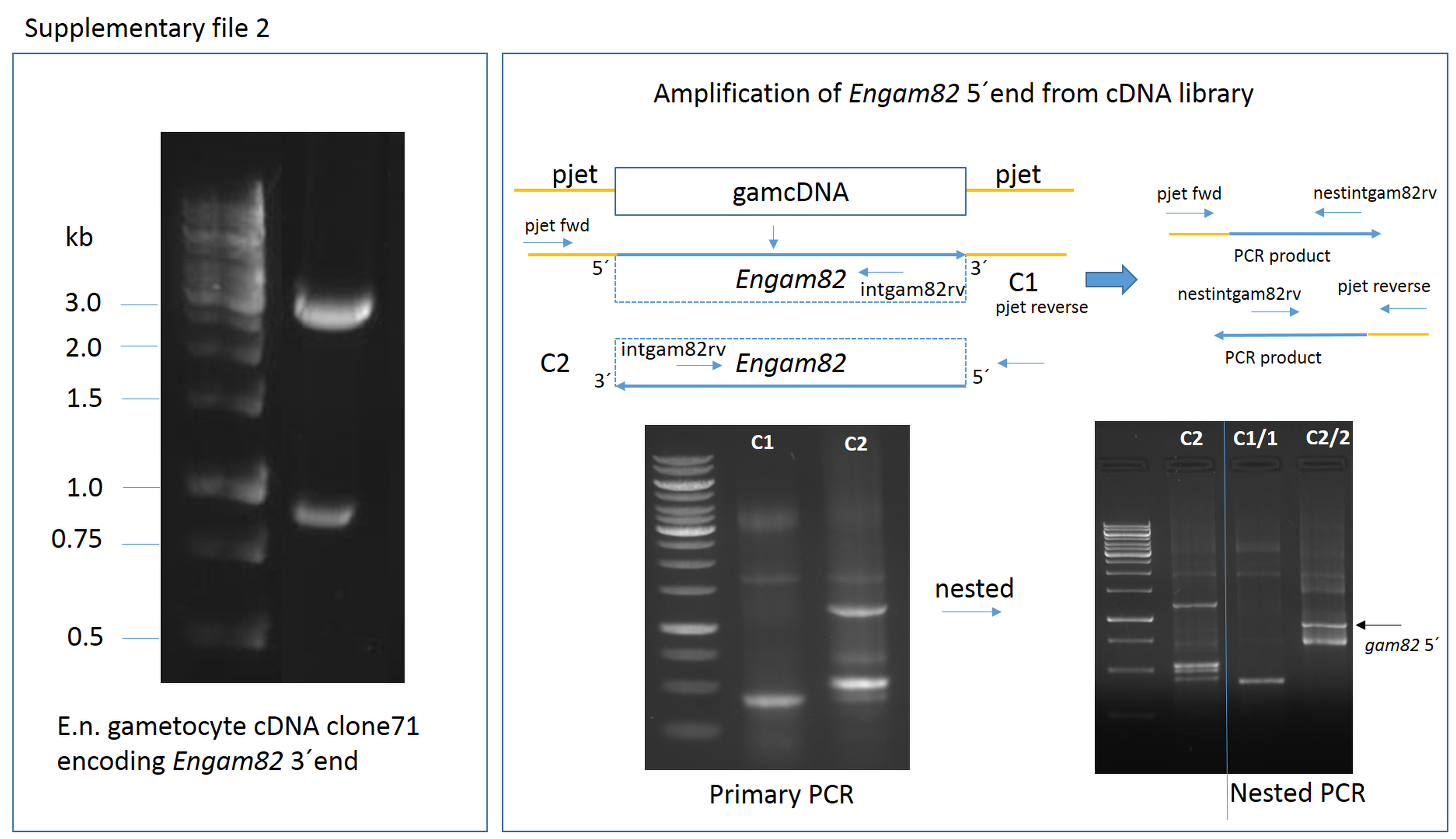

Supplement: Supplementary file 1 — SF1 A) DNA sequence of gt2Engam56_2; B) Sequence alignment of representative EnGAM56_2 and variant version gt2EnGAM56_2; C) Alignment of 140 amino acids (translated DNA) of EfalGAM56_2 and EnGAM56_2; D) DNA sequence of gt2Engam56_1; E) Sequence alignment of representative EnGAM56_1 and variant version gt2EnGAM56_1; F) Comparison of different repeat length in GAM56_2 homologs in two closely related avian Eimeria species; G) Sequence alignment of GAM56 proteins; H) Sequence alignment of GAM82 proteins. SF 2: Obtaining Engam82 sequence from cDNA library; SF 3: Contigs_cov_below_40; SF 4: Contigs_cov_above_40. [file parasite-24-50-s1.zip › parasite170085-1-olm/SF2.png]
